# Actomyosin-dependent assembly of the mechanosensitive machinery from adherens junctions triggers actin polymerization and organization

**DOI:** 10.1126/sciadv.ady4863

**Published:** 2026-01-01

**Authors:** Aurélie Favarin, Rayan Said, Hong Wang, Emilie Zeine, Amaury Pierrard, Julien Pernier, Hemalatha Narassimprakash, Stéphane Roméro, Alexis M. Gautreau, René-Marc Mège, Christophe Le Clainche

**Affiliations:** ^1^ Université Paris-Saclay, CEA, CNRS, Institute for Integrative Biology of the Cell (I2BC), 91198 Gif-sur-Yvette, France.; ^2^ Laboratoire de Biologie Structurale de la Cellule, CNRS, Ecole Polytechnique, Institut Polytechnique de Paris, Palaiseau, France.; ^3^ Université Paris-Cité, CNRS, Institut Jacques Monod, Paris, France.

## Abstract

Cells rely on cadherin-based adherens junctions (AJs) to form cohesive tissues. To establish contact, cells generate pushing forces through branched actin polymerization mediated by the actin-related protein 2/3 (Arp2/3) complex, followed by the reinforcement of mechanosensitive AJs in response to actomyosin contractility. To investigate how AJ proteins coordinate these events, we combined kinetic assays of actin polymerization, single actin filament observation in total internal reflection fluorescence microscopy, and in vitro reconstitution of AJ mechanosensitivity. Our findings show that actomyosin contractility alone is sufficient to trigger the hierarchical assembly of the AJ mechanosensitive proteins α-catenin, vinculin, and vasodilator-stimulated phosphoprotein (VASP). Once assembled, these proteins act synergistically to promote actin filament nucleation, elongation, and bundling. The α-catenin-vinculin-VASP machinery inhibits Arp2/3-mediated actin branching and instead promotes the myosin-dependent assembly of actin bundles. Together, these results reveal how AJs integrate actin assembly, actomyosin contractility, and mechanosensitivity in a feedback loop.

## Teaser

In vitro reconstitution shows how mechanosensitive adherens junction proteins link actomyosin contractility to actin assembly.

## INTRODUCTION

The formation and dynamics of tissues during the development of multicellular organisms, their constant repair during wound healing and tissue regeneration, and their adaptation to mechanical perturbations require adaptive adhesion between cells. Cadherin-dependent adherens junctions (AJs) are key cell-cell adhesion structures that contribute substantially to tissue integrity and maintenance (*1*–*3*). Beyond passive adhesion, they function as mechanosensors that mediate adaptive responses to mechanical force fluctuations in tissues (*4*–*6*).

During the formation and repair of AJs, cells extend membrane protrusions driven by the growth of branched actin networks mediated by the actin-related protein 2/3 (Arp2/3) complex to bring cadherins into contact (*7*–*10*). Once formed, AJs mature into stable structures that assemble actomyosin (*11*–*13*). Deciphering the molecular mechanisms that govern the transition between actin networks of different dynamics and architecture is key to understand the formation and constant remodeling of AJs. However, the molecular mechanisms underlying actin dynamics associated with AJs are poorly understood.

The interconnected proteins β-catenin, α-catenin, vinculin, and vasodilator-stimulated phosphoprotein (VASP) mechanically couple cadherins to the actin cytoskeleton (*11*, *14*–*18*). The N-terminal dimerization domain of α-catenin dissociates into a monomer upon interaction with β-catenin. The central region and C-terminal part of α-catenin contain a vinculin-binding site (VBS) and an actin filament-binding domain (ABD), respectively. Last, the actin regulator VASP interacts with an FPPPP motif in the proline-rich linker of vinculin (*19*). Along this force transmission pathway, the α-catenin-vinculin interaction is the main mechanosensitive switch. Cell-based experiments suggest that force applied to the ABD of α-catenin relieves its autoinhibition, exposing the VBS and promoting vinculin binding to reinforce AJs (*20*–*24*). Stretching the central domain of a single α-catenin molecule under a 5-pN force exposes a cryptic VBS that binds vinculin (*25*, *26*). In addition, α-catenin strengthens its bond with actin under force through a catch bond mechanism (*27*–*30*). Although the abovementioned cellular observations and single-molecule stretching studies support a mechanism for α-catenin–mediated mechanosensing, it remains to be demonstrated in vitro whether actomyosin-generated forces can trigger the hierarchical assembly of α-catenin, vinculin, and VASP.

Several cellular observations suggest that the proteins linking cadherins to actin also regulate actin polymerization in a myosin-dependent manner. Supporting this, knockdown of nonmuscle myosin II isoforms (NMIIA and NMIIB) reduces the amount of polymerized actin at AJs (*31*). This process involves the recruitment of vinculin to AJs through the VBS of α-catenin and depends on Mena/VASP binding to the FPPPP motif within vinculin’s proline-rich linker (*31*, *32*). At the molecular level, α-catenin, vinculin, and VASP have ABDs that act on actin polymerization and organization in different ways. Vinculin nucleates actin filaments capped at their barbed ends and bundles them (*33*, *34*), with bundling requiring the dimerization of its ABD called Vt (*35*, *36*). α-Catenin also inhibits barbed-end elongation via its ABD (*35*). In addition, tetrameric proteins from the VASP family combine nucleation, barbed-end elongation, and bundling functions (*37*–*40*). However, the individual actin polymerization activities of these AJ proteins do not predict the outcome of their combined action.

To determine the activity of the α-catenin-vinculin-VASP machinery, we combine measurements of actin polymerization kinetics using fluorescence spectroscopy with single actin filament observations using total internal reflection fluorescence (TIRF) microscopy. Our results show that α-catenin, vinculin, and VASP coordinate their activities to stimulate the nucleation and elongation of linear actin filaments from profilin-actin and cross-link them into bundles. We also reconstitute actin assembly by this machinery on a micropatterned surface that mimics the two-dimensional architecture of the apical face of epithelial tissue. In this assay, applying actomyosin force to α-catenin immobilized in micropatterns triggers the recruitment of vinculin and VASP, which amplifies and spatially propagates actin assembly. Last, the α-catenin-vinculin-VASP machinery suppresses Arp2/3-mediated actin branching and instead promotes the myosin-dependent assembly of actin bundles. Collectively, these findings highlight how AJs coordinate actin assembly, contractility, and mechanosensitive responses.

## RESULTS

### In vitro assembly of the α-catenin-vinculin-VASP machinery

**Fig. 1.**
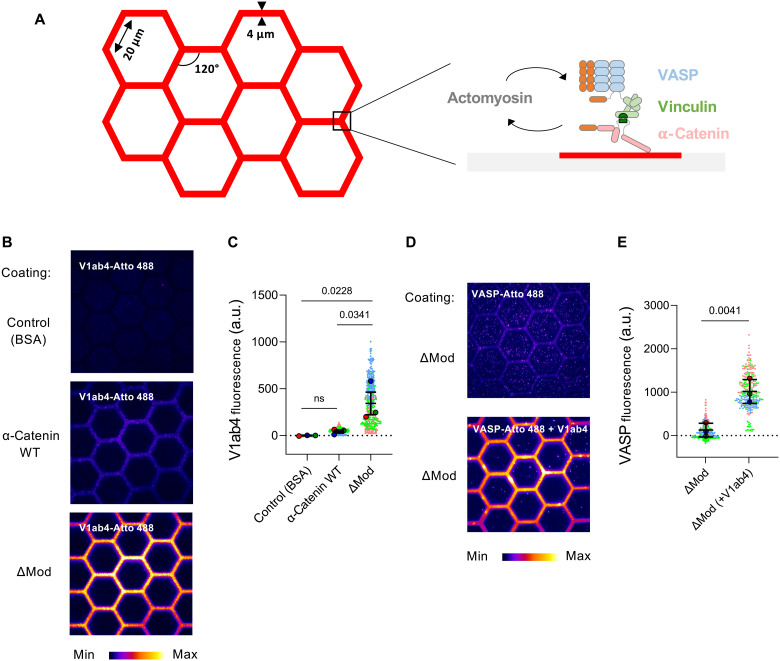
In vitro assembly of the α-catenin-vinculin-VASP machinery. (**A**) Basic principle of the in vitro microscopy assay used to study the formation and the activity of the α-catenin-vinculin-VASP machinery on a micropatterned surface. (**B**) Atto 488–labeled V1ab4 (1 μM) was added to micropatterned surfaces coated with either BSA (1%), α-catenin WT, or ΔMod (1 μM) and imaged (fire LUT from ImageJ) after 5 min using TIRF microscopy. Scale bar, 20 μm. (**C**) Quantification of Atto 488–labeled V1ab4 fluorescence intensity along the edges of the micropatterned hexagons in the conditions shown in (B). Each data point is one side of a hexagon. Data are the means ± SEM, *n* = 200 to 300 sides per condition, *N* = 3. a.u., arbitrary units. (**D**) Atto 488–labeled VASP (1 μM, top panel), or a mix of V1ab4/VASP (1:1, 1 μM final, bottom panel), was added to a micropatterned surface coated with ΔMod (1 μM) and imaged (fire LUT from ImageJ) after 5 min using TIRF microscopy. Scale bar, 20 μm. (**E**) Quantification of Atto 488–labeled VASP fluorescence intensity along the edges of the micropatterned hexagons in the conditions shown in (D). Each data point is one side of a hexagon. Data are the means ± SEM, *n* = 300 to 400 sides per condition, *N* = 3. [(C) and (E)] Each color represents an independent experiment. *P* values were obtained using a one-tailed unpaired *t* test comparing the mean values.

To understand the mechanisms underlying actin assembly by the mechanosensitive machinery of AJs, composed of α-catenin, vinculin, and VASP, we reconstituted this process on a micropatterned surface (Fig. 1A). We chose a honeycomb design coated with α-catenin. While this system does not fully reproduce the features of AJs connecting neighboring cell membranes or the dynamic architecture of cells within a tissue, it shares some key characteristics with the apical organization of AJs. It allows us to probe the spatiotemporal propagation of α-catenin partner recruitment along hexagonal edges in response to actomyosin contractility.

Before investigating the mechanosensitivity of the α-catenin-vinculin-VASP machinery, we first sought to determine its actin polymerization activity independently of actomyosin-generated forces. Given that the assembly of this machinery depends on force, its actin polymerization activity must be assessed using constructs that bind constitutively. In α-catenin, the VBS in the MI domain is masked by the adjacent MII and MIII domains. Vinculin is similarly autoinhibited through an intramolecular interaction between its head domain (Vh), which binds the VBS of α-catenin, and Vt, which binds actin. To bypass these regulations, we used the α-catenin ΔMod construct, which lacks MII and MIII, thereby constitutively exposing its VBS (fig. S1A) (*22*). We also used a vinculin construct, V1ab4, carrying point mutations that reduce autoinhibition (fig. S1B) (*41*). VASP interacts with vinculin regardless of autoinhibitory regulation (fig. S1C).

We developed a binding assay in which a micropatterned surface coated with bovine serum albumin (BSA), α-catenin [wild type (WT)], or ΔMod is incubated with fluorescent Atto 488–labeled V1ab4. Our results show that V1ab4 binds constitutively to ΔMod but not to α-catenin WT or BSA (Fig. 1, B and C). Moreover, Atto 488–labeled VASP is recruited to a ΔMod-coated surface only in the presence of V1ab4, which enables the hierarchical assembly of α-catenin, vinculin, and VASP (Fig. 1, D and E). While α-catenin and vinculin are characterized as dimers, VASP forms a tetramer. Consequently, their assembly is expected to give rise to a protein network rather than a finite-sized complex. For this reason, in this study, we refer to α-catenin-vinculin-VASP as a machinery rather than a complex.

### α-Catenin, vinculin, and VASP synergize to nucleate actin filaments

To investigate how these three proteins influence actin dynamics, we first assessed their effects on the kinetics of pyrene-labeled actin polymerization using fluorescence spectroscopy. This method enables the rapid screening of multiple conditions and the detection of subtle variations. Conveniently, actin-binding proteins such as VASP and vinculin display ionic strength–dependent activities, promoting nucleation at low ionic strength while retaining the ability to modulate barbed-end elongation at higher levels (*33*, *40*). This enables experimental uncoupling of their activities.

**Fig. 2.**
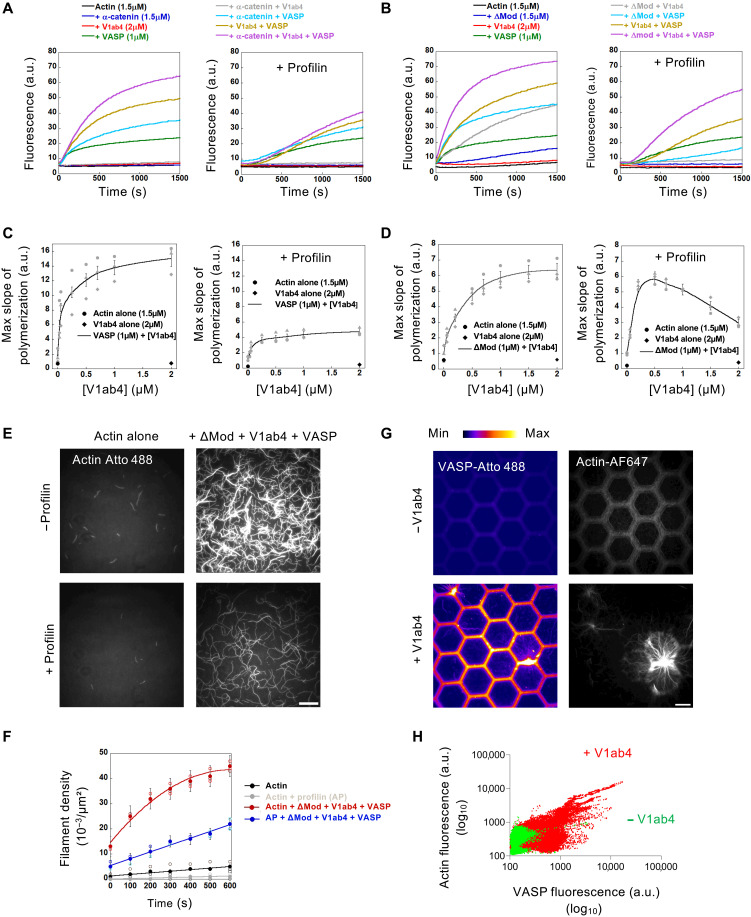
α-Catenin, vinculin, and VASP synergize to nucleate actin filaments. (**A** and **B**) The spontaneous nucleation of 1.5 μM G-actin (10% pyrene-labeled) was measured in the presence of the indicated combinations of proteins in the absence (left) or presence of 10 μM profilin (right) in 25 mM KCl. (A) With α-catenin WT and (B) with α-catenin ΔMod. (**C** and **D**) The maximal polymerization rate of 1.5 μM G-actin (10% pyrene-labeled) is plotted against increasing V1ab4 concentrations under the indicated conditions in low-salt buffer (25 mM KCl). Gray data points (discs, diamonds, and triangles) represent three independent experiments under identical conditions. The black line connects the means ± SEM. (**E**) Single actin filaments observed using TIRF microscopy in the presence of 0.8 μM actin (10% Atto 488–labeled) alone and supplemented with ΔMod (1 μM), V1ab4_,_ (1 μM), and VASP (0.6 μM) in a low-salt buffer (25 mM KCl) in the absence (top) or presence (bottom) of profilin (5 μM). See movie S1. (**F**) Quantification of filament density as a function of time from the experiments shown (E). Open symbols denote data from three independent experiments under identical conditions. Closed circles mark mean values, with error bars showing SEM. (**G**) Assembly of 1 μM actin (2% Alexa Fluor 647N–labeled) in the presence of 5 μM profilin on a micropatterned surface incubated with either a mix of ΔMod/Atto 488-VASP (−V1ab4) at 1.6 μM (1:1) (top) or a mix of ΔMod/V1ab4/Atto 488-VASP (+V1ab4) at 1.6 μM (1:1:1) (bottom). VASP (fire LUT) and actin (grays). Images are acquired using TIRF microscopy at 50 min. Scale bar, 20 μm. See movie S2. (**H**) Quantification of (G) showing actin fluorescence versus VASP fluorescence within micropatterns. Each data point is a single pixel from *n* = 50 to 60 edges per condition, *N* = 3.

To investigate the effect of combinations of α-catenin, vinculin, and VASP on actin nucleation, we first tested whether these proteins could stimulate spontaneous actin polymerization under low ionic strength conditions. We observed the strongest stimulation of actin assembly in the presence of the three proteins α-catenin (WT and ΔMod), V1ab4, and VASP (Fig. 2, A and B, left panels). Although the apparent nucleation activity observed at 25 mM KCl decreases with higher ionic strength, it persists at a substantial level at 100 mM KCl (fig. S2, A and B). Of the three proteins tested alone, only VASP shows significant activity, while α-catenin (WT and ΔMod) and V1ab4 exhibit little to no activity (Fig. 2, A and B, left panels). When combined, V1ab4 enhances VASP activity (Fig. 2, A and B, left panels), with a clear dose-dependent effect (Fig. 2C, left, and fig. S3A). The combination of V1ab4 and α-catenin ΔMod stimulates actin assembly, likely due to V1ab4 activation by the exposed VBS of ΔMod (Fig. 2B, left), in a dose-dependent manner (Fig. 2D, left, and fig. S3C). The lack of stimulation of V1ab4 by autoinhibited α-catenin WT further confirms the requirement for VBS exposure (Fig. 2A, left). VASP and α-catenin (WT or ΔMod), which are not known to interact, synergistically promote actin assembly, suggesting indirect cooperation (Fig. 2, A and B, left panels).

In cells, most polymerizable actin is bound to profilin, which prevents spontaneous nucleation and allows spatial and temporal regulation by specific machineries. Profilin also serves as a cofactor for regulators like VASP and formins (*38*, *42*). In the presence of profilin, actin stimulation remains maximal with all three proteins at 25 mM KCl (Fig. 2, A and B, right panels) and persists at 50 mM KCl (fig. S2, C and D). V1ab4 synergizes with VASP (Fig. 2, A and B, right panels) in a dose-dependent manner (Fig. 2C, right, and fig. S3B). α-Catenin ΔMod also enhances V1ab4 activity more than α-catenin WT (Fig. 2, A and B, right panels), and this enhancement is dose-dependent (Fig. 2D, right, and fig. S3D). Last, a dose-response analysis of the effect of α-catenin ΔMod, vinculin V1ab4, and VASP (kept at a 1:1:1 stoichiometry) on actin polymerization reveals that the effect becomes significant at 250 nM in the presence of 1.5 μM actin and 10 μM profilin, which corresponds to a ΔMod:V1ab4:VASP:actin ratio of 1:1:1:6 (fig. S4, A and B). Together, these results indicate that the α-catenin-vinculin-VASP machinery nucleates actin filaments and overcomes profilin inhibition.

We also used TIRF microscopy to observe single actin filament density, which reflects nucleation, and confirmed that combining V1ab4, ΔMod, and VASP enhances nucleation, even in the presence of profilin, although less efficiently (Fig. 2, E and F, and movie S1). To study the dynamics and organization of actin filaments nucleated by the preassembled machinery from structures mimicking the belt-like arrangement of AJs, we preincubate Alexa Fluor 568–labeled α-catenin ΔMod, V1ab4, and Atto 488–labeled VASP, immobilize them on a honeycomb micropatterned surface (Fig. 1A), and add Alexa Fluor 647–labeled actin and profilin. The three proteins form surface-bound patches, visible via VASP fluorescence, that serve as platforms for actin growth (Fig. 2G and movie S2). Without V1ab4, VASP is not recruited and actin assembly is minimal. Quantification confirms that VASP recruitment to ΔMod via V1ab4 is essential (Fig. 2H and fig. S5). Actin polymerization is not required for the formation of these patches, as these can be observed in the absence of actin and subsequently drive actin polymerization when actin is added later (fig. S6). The heterogeneous polymerization pattern, with explosive events occurring specifically at VASP-enriched sites (Fig. 2G), supports a role for VASP clustering mediated by vinculin and α-catenin. These findings suggest that VASP triggers actin polymerization efficiently only above a threshold cluster size.

### α-Catenin, vinculin, and VASP cooperate to stimulate actin filament elongation

**Fig. 3.**
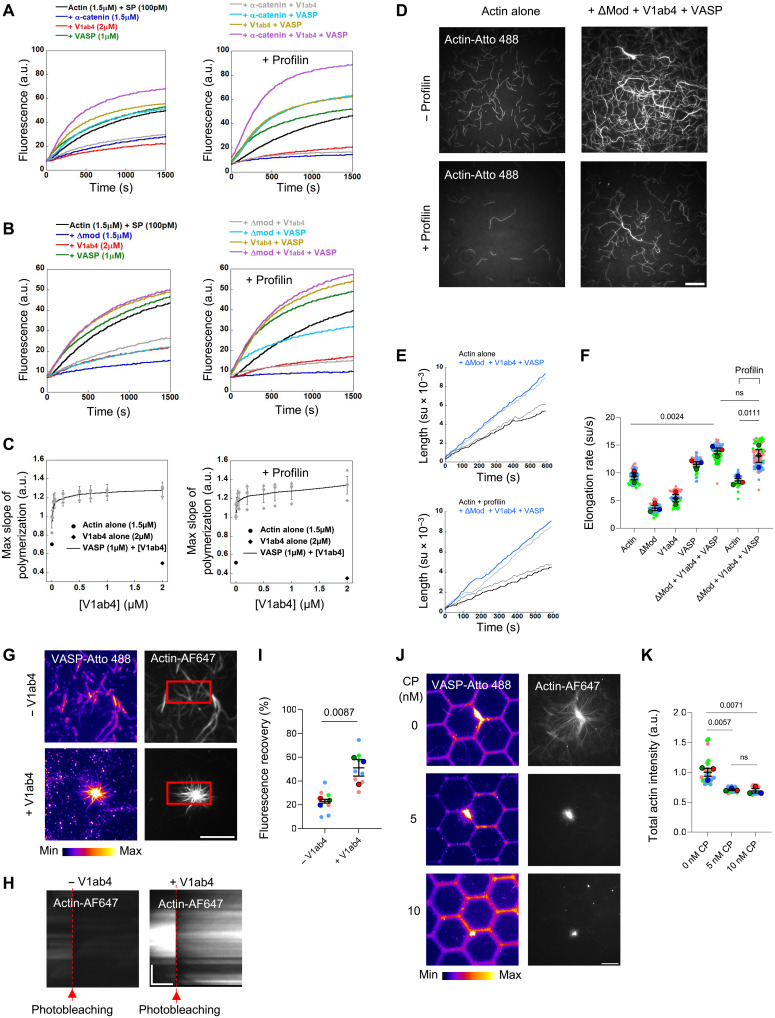
α-Catenin, vinculin, and VASP cooperate to stimulate actin filament elongation. (**A** and **B**) Barbed-end elongation of 1.5 μM G-actin (10% pyrene) from 100 pM spectrin-actin seeds with indicated proteins, without (left) or with 10 μM profilin (right), in 100 mM KCl. α-Catenin WT (A) and ΔMod (B). (**C**) Maximal barbed-end elongation of 1.5 μM G-actin (10% pyrene) from 100 pM spectrin-actin seeds versus V1ab4 concentration with 1 μM VASP, without (left) or with profilin (right), in 100 mM KCl. Gray symbols show three independent experiments; the black line connects the means ± SEM. (**D**) Assembly of 0.8 μM actin (10% Atto 488) alone or with ΔMod (1 μM), V1ab4 (1 μM), and VASP (0.6 μM), without (top) or with profilin (5 μM; bottom) in 100 mM KCl. TIRF microscopy. See movie S3. (**E**) Single filaments elongating as in (D), without (top) or with profilin (bottom). Same-colored traces show independent experiments. su, subunits. (**F**) Barbed-end elongation rate between pauses. Means ± SEM, *n* = 10 to 60 filaments per condition, *N* = 3. ns, not significant. (**G**) Assembly of 1 μM actin (2% Alexa Fluor 647N) with 5 μM profilin on micropatterns, incubated with ΔMod/Atto 488-VASP (−V1ab4, 1.6 μM, 1:1, top) or ΔMod/V1ab4/Atto 488-VASP (+V1ab4, 1.6 μM, 1:1:1, bottom) before FRAP (square). Scale bar, 20 μm. See movie S4. (**H**) Kymographs of FRAP regions in (G). Scale bars, 5 μm (vertical) and 500 s (horizontal). (**I**) Fluorescence recovery in (G) after 25 min. (**J**) Assembly of 5 μM actin (2% Alexa Fluor 647N) with 20 μM profilin on micropatterns with ΔMod/V1ab4/Atto 488-VASP (1.6 μM, 1:1:1) and 0, 5, and 10 nM CP. Scale bar, 20 μm. See movie S5 (same conditions, different experiment). (**K**) Actin intensity at 20 min in (J). Means ± SEM, *n* = 30 images per condition, *N* = 3. VASP (fire LUT) and actin (gray). *P* values from a one-tailed unpaired *t* test.

We assess the effect of α-catenin, vinculin, and VASP on actin elongation using kinetic assays with spectrin-actin seeds, which are short filaments that elongate at their barbed end, to minimize the contribution of spontaneous nucleation. To further reduce VASP nucleation activity, which is favored at low ionic strength (*40*), assays are performed at 100 mM KCl. We confirm previous findings that VASP slightly promotes barbed-end elongation of actin filaments, while α-catenin (WT and ΔMod) and V1ab4 inhibit elongation, both with and without profilin (Fig. 3, A and B) (*33*, *38*, *41*, *43*). We find that VASP protects actin filament barbed-end elongation from α-catenin (Fig. 3A, left) but is less effective against ΔMod (Fig. 3B, left), suggesting that ΔMod more readily exposes its ABD. VASP also protects barbed ends from the capping activity of V1ab4 (Fig. 3, A to C, left panels), even in the presence of profilin (Fig. 3, A to C, right panels, and fig. S7, A and B). This protective effect on barbed ends can be readily explained by the hierarchy of binding affinities of the three proteins for actin barbed ends. Using kinetic assays, we estimate the affinities of α-catenin ΔMod and vinculin V1ab4 for barbed ends to be 0.35 and 1.26 μM, respectively (fig. S8, A to C), which are too weak to compete with VASP, whose known affinity for barbed ends is 9.2 nM (*39*).

To confirm these observations, we examined the effect of α-catenin ΔMod, V1ab4, and VASP on single actin filament elongation using TIRF microscopy (Fig. 3D and movie S3). Quantification shows that this combination also enhances barbed-end elongation with and without profilin (Fig. 3, E and F).

VASP clusters promote processive-like actin elongation (*38*). To assess whether actin filaments elongate processively from ΔMod-V1ab4-VASP clusters immobilized in micropatterns, we performed fluorescence recovery after photobleaching (FRAP) on actin (Fig. 3G and movie S4). Kymographs show that both newly polymerizing and bleached regions from ΔMod-V1ab4-VASP clusters remain stationary, ruling out processive elongation (Fig. 3H). Without V1ab4, actin polymerizes less efficiently, as reflected by lower initial fluorescence and weak recovery after photobleaching (Fig. 3I). Although human VASP dissociates rapidly from barbed ends (*39*), transient interactions with VASP clusters may still protect them. Supporting this, increasing concentrations of the well-characterized capping protein (CP) fail to fully block actin filament growth, resulting in a network of short, VASP-decorated filaments (Fig. 3, J and K, and movie S5).

### α-Catenin, vinculin, and VASP synergize to bundle actin filaments

To determine whether combinations of α-catenin, vinculin, and VASP promote actin bundling, we combine light scattering with fluorescence imaging (fig. S9). Light scattering reveals minimal bundling by V1ab4, α-catenin (WT or ΔMod), and VASP alone but a marked increase when the proteins are combined (fig. S9, A to D). Substituting α-catenin WT with ΔMod further enhances bundling with V1ab4 and VASP (fig. S9, C and D), highlighting the contribution of the α-catenin-vinculin complex to actin filament cross-linking. Fluorescence microscopy of actin filaments recovered from the light scattering assays confirms the presence of thin bundles with individual proteins or pairs, whereas the combination of ΔMod, V1ab4, and VASP produces thicker actin bundles (fig. S9E). These findings corroborate the actin bundling previously observed by TIRF microscopy under the same conditions (Figs. 2E and 3D).

### Vinculin associates with α-catenin in response to actomyosin activity

**Fig. 4.**
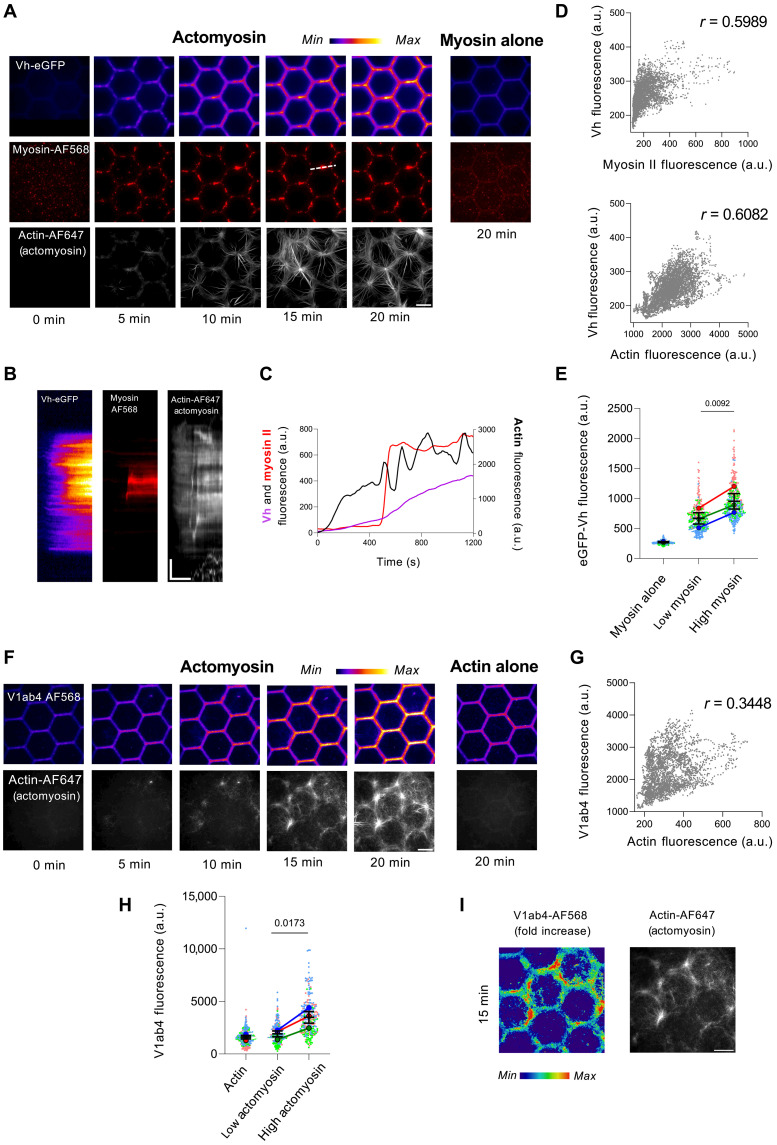
Vinculin associates with α-catenin in response to actomyosin contractility. (**A**) Time lapse of Vh-eGFP (100 nM) and Alexa Fluor 568-myosin II (50 nM) on α-catenin WT–coated micropatterns (10 μM during coating), with actin (2.4 μM, 2% Alexa Fluor 647N) and profilin (10 μM) on the left or without actin and profilin (right, myosin alone). Vh-EGFP (fire LUT), Alexa Fluor 568-myosin II (red), and Alexa Fluor 647N-actin (gray). Scale bar, 20 μm. See movie S6. (**B**) Kymographs of Vh-eGFP, Alexa Fluor 568-myosin, and Alexa Fluor 647N-actin recruitment to an α-catenin WT surface from (A). Scale bar: vertical (l), 5 μm; horizontal (t), 500 s. (**C**) Plot profiles from (B). (**D**) From (A), Vh-eGFP versus myosin (top) and actin (bottom) fluorescence intensities within micropatterns after 10 min. (**E**) Vh-eGFP fluorescence in α-catenin WT patterns from (A) after 20 min. With actomyosin, Vh-eGFP is measured in myosin-rich (high) and adjacent myosin-poor (low) regions. (**F**) Time lapse of Alexa Fluor 568-V1ab4 (100 nM) in α-catenin WT micropatterns with 2.4 μM actin (2% Alexa Fluor 647N) and 10 μM profilin without (right) or with 50 nM myosin II (left). V1ab4 (fire LUT) and actin (gray). Scale bar, 20 μm. See movie S7. (**G**) From (F), V1ab4 plotted against actin fluorescence intensities within micropatterns after 15 min. (**H**) V1ab4 recruitment in α-catenin WT patterns from (F) after 15 min. With actomyosin, V1ab4 was measured in actomyosin-rich (high) and adjacent actomyosin-poor (low) regions. (**I**) Fold increase in V1ab4 fluorescence (15-min image divided by 5-min preactomyosin image). The 15-min actin image, identical to (F), is shown for comparison. V1ab4 (physics LUT) and actin (gray). Scale bar, 20 μm. [(E) and (H)] Data are the means ± SEM, *n* = 60 to 300 per condition, *N* = 3. Each color indicates an independent experiment. *P* values from a one-tailed paired *t* test. [(D) and (G)] Each point is a pixel. Experiments were repeated three times with similar results.

α-Catenin ΔMod displays higher affinity for vinculin than α-catenin WT (Fig. 1B), suggesting that actomyosin-generated force may be sufficient to unfold α-catenin WT and expose its VBS. To test the hypothesis that myosin II pulls on actin filaments linked to the α-catenin–coated surface to expose α-catenin’s VBS and therefore promote vinculin binding, we examined the colocalization of Alexa Fluor 568–labeled myosin II, Alexa Fluor 647–labeled actin, and the vinculin head fused to enhanced green fluorescent protein (eGFP; Vh-eGFP) (*44*), which lacks the C-terminal actin-binding domain (Vt), on a micropatterned surface coated with α-catenin WT. Myosin assembles into micrometric structures resembling myosin II minifilaments that move along actin filaments. Their activity drives the contraction of actin filament foci where myosins converge to form patches, triggering Vh-eGFP recruitment (Fig. 4A and movie S6). In contrast, myosin II and Vh-eGFP accumulation is absent in a control without actin (Fig. 4A). Kymograph analysis reveals the concomitant formation of a myosin II patch together with actin recruitment, contraction of actin filaments toward the center, and Vh-eGFP recruitment (Fig. 4, B and C). The role of myosin in actin contraction and in Vh-eGFP recruitment at the α-catenin surface is further supported by the pairwise correlation of Vh-eGFP fluorescence intensity with that of myosin-Alexa Fluor 568 and actin-Alexa Fluor 647 (Fig. 4D), as well as by the stronger recruitment of Vh-eGFP on α-catenin WT–coated patterns associated with myosin patches (high myosin), compared to regions devoid of myosin patches (low myosin) (Fig. 4E).

Similarly, Alexa Fluor 568–labeled V1ab4, which contains the Vt domain, is recruited to α-catenin WT–coated patterns in the presence of actomyosin compared with conditions containing actin alone (Fig. 4F and movie S7). Quantification shows that the correlation between V1ab4-Alexa Fluor 568 and actin-Alexa Fluor 647 in the presence of myosin is lower than that previously observed for Vh-eGFP (Fig. 4G). Nevertheless, V1ab4 recruitment is significantly enhanced on α-catenin WT–coated patterns undergoing actomyosin contraction compared with regions of low actomyosin activity (Fig. 4H). To better visualize actomyosin-dependent V1ab4 recruitment, we calculated the fold increase in local V1ab4 fluorescence by dividing the 15-min image by the 5-min preactomyosin image. The processed image confirms enhanced V1ab4 binding in α-catenin–coated regions enriched with actomyosin at 15 min (Fig. 4I).

### Actomyosin-driven assembly of the α-catenin-vinculin-VASP complex promotes actin polymerization and establishes feedback to actomyosin

**Fig. 5.**
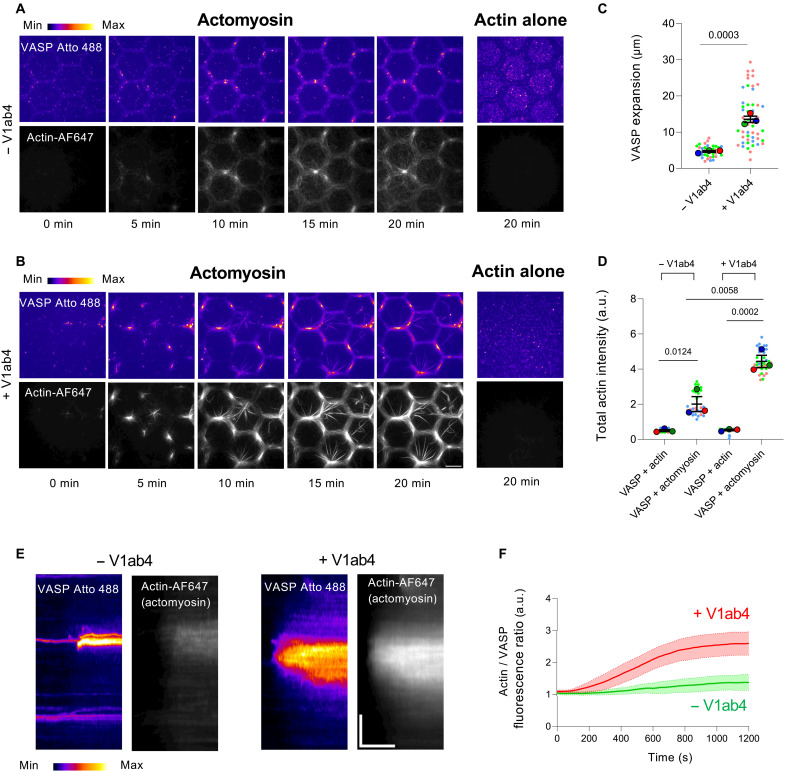
Actomyosin-driven assembly of the α-catenin-vinculin-VASP complex promotes actin polymerization and establishes feedback to actomyosin. (**A** and **B**) Left: Time-lapse images of Atto 488–labeled VASP (100 nM) recruitment to a micropatterned surface coated with 10 μM α-catenin WT in the presence of 2.4 μM actin (2% Alexa Fluor 647N–labeled), 10 μM profilin, and 50 nM myosin II, either without (A) or with (B) 100 nM V1ab4. VASP shown in fire LUT (ImageJ) and actin in gray. Scale bar, 20 μm. See movie S8. Right: TIRF microscopy images at *t* = 20 min of the same conditions as above without myosin, showing VASP recruitment to α-catenin–coated micropatterns in the absence (A) or presence (B) of V1ab4. (**C**) Quantification of the expansion of VASP-enriched domains along α-catenin WT–coated patterns in response to actomyosin contraction. Data are the means ± SEM, *n* = 10 to 20 patches per condition, *N* = 3. (**D**) Quantification of total actin intensity after 20 min under the conditions described in (A) and (B). Data are the means ± SEM, *n* = 30 images per condition, *N* = 3. (**E**) Kymographs showing VASP expansion along a hexagon edge. Scale bars: vertical, 5 μm; horizontal, 500 s. (**F**) Kinetics of the actin/VASP fluorescence intensity ratio under the conditions described in (A) and (B) (left panels). Data are the means ± SEM. For +V1ab4, *n* = 211, *N* = 3; for −V1ab4, *n* = 107, *N* = 3. [(C) and (D)] Each color represents an independent experiment. *P* values were calculated using a one-tailed unpaired *t* test comparing mean values.

We then tested the hypothesis that the α-catenin-vinculin-VASP machinery assembles in response to actomyosin contraction and, in turn, provides feedback to actomyosin by promoting the polymerization of myosin-associated actin filaments. We first show that actomyosin activity promotes the recruitment of Atto 488-VASP to α-catenin WT–coated micropatterns in the presence of V1ab4. VASP is more strongly recruited to actomyosin-rich regions than to areas with low actomyosin or actin alone (Fig. 5B and movie S8), and this recruitment is markedly reduced without V1ab4, indicating a requirement for vinculin (Fig. 5A and movie S8). Time-lapse imaging reveals that small VASP clusters initially associate with actomyosin outside α-catenin–coated regions and then migrate toward these areas, where they become immobilized and elongate along the pattern (movie S8). These domains extend up to 30 μm with V1ab4 but fail to spread in its absence (Fig. 5, A to C), suggesting a cooperative mechanism that promotes local complex formation. Last, both actin content (Fig. 5D) and actin/VASP ratios (Fig. 5, E and F) are increased in α-catenin–coated regions with V1ab4, consistent with a positive feedback loop between actomyosin contractility and actin assembly via the mechanosensitive α-catenin-vinculin-VASP machinery.

### The α-catenin-vinculin-VASP machinery inhibits Arp2/3-mediated branching and instead promotes myosin-dependent actin bundle assembly

α-Catenin has been shown to inhibit Arp2/3-mediated branched actin assembly (*17*), while vinculin can reorganize branched networks into bundles and inhibit Arp2/3-dependent cell migration and proliferation (*45*, *46*). Myosin II also disassembles and remodels actin networks (*47*). These findings led us to investigate the interplay between actomyosin, the α-catenin-vinculin-VASP machinery, and Arp2/3.

**Fig. 6.**
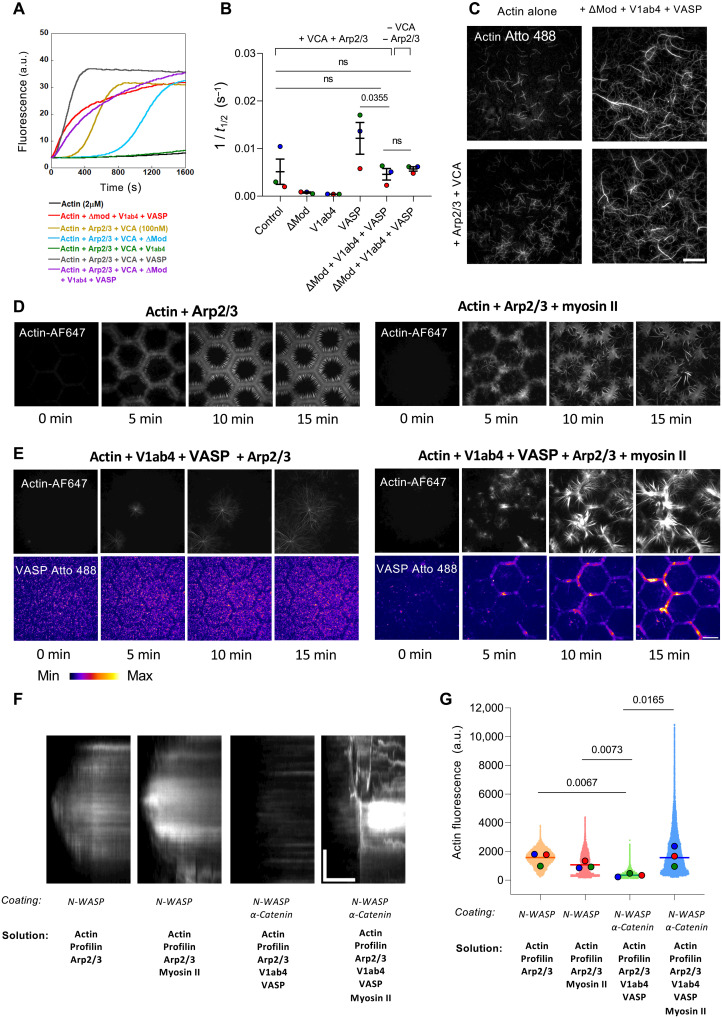
The α-catenin-vinculin-VASP machinery inhibits Arp2/3-mediated actin branching and instead promotes the myosin-dependent assembly of actin bundles. (**A**) Actin polymerization kinetics measured with 2 μM G-actin (10% pyrene-labeled) and indicated combinations of 50 nM Arp2/3, 100 nM VCA, 5 μM ΔMod, 5 μM V1ab4, and 3 μM VASP in 50 mM KCl. (**B**) Inverse polymerization half-time (1/*t*_1/2_) from (A). Different colors indicate three independent experiments. Data are the means ± SEM, *N* = 3. (**C**) Single actin filaments in TIRF with 0.8 μM actin (10% Atto 488–labeled) and indicated combinations of 50 nM Arp2/3, 100 nM VCA, 1 μM ΔMod, 1 μM V1ab4, and 0.6 μM VASP (images taken at 450 s). Scale bar, 20 μm. See movie S9. (**D**) Time lapse of 2.4 μM actin (2% Alexa Fluor 647N) with 10 μM profilin on N-WASP–coated micropatterns (100 nM), supplemented with 50 nM Arp2/3 alone (left) or Ap2/3 and 50 nM myosin II (right). (**E**) Time lapse of 2.4 μM actin (2% Alexa Fluor 647N) with 10 μM profilin, 100 nM V1ab4, and 100 nM VASP-Atto 488 on micropatterned surfaces coated with 100 nM N-WASP and 10 μM α-catenin WT, supplemented with 50 nM Arp2/3 alone (left) or Arp2/3 and 50 nM myosin II (right). [(D) and (E)] VASP (fire LUT) and actin (gray). Scale bar, 20 μm. See movie S10. (**F**) Kymographs of actin along the edge of a hexagon from (D) and (E). Scale bar: vertical (l), 5 μm; horizontal (t), 500 s. (**G**) Actin intensity along hexagon edges after 20 min from (D) and (E). The scatterplot shows all pixels from three independent experiments; larger points indicate the median of each repeat. *n* = 35,000 pixels per condition, *N* = 3. (B) and (G) *P* values from a one-tailed unpaired *t* test comparing means (B) and medians (G).

Kinetic assays show that ΔMod and V1ab4 individually inhibit actin assembly driven by Arp2/3 activated by the verprolin-central-acidic (VCA) domain of neural Wiskott-Aldrich syndrome protein (N-WASP), while VASP slightly reduces the lag phase but has little impact on the polymerization rate (Fig. 6, A and B). Combining ΔMod, V1ab4, and VASP stimulates actin polymerization equally with or without Arp2/3 and VCA, indicating that their joint activity overrides the branched assembly pathway (Fig. 6, A and B). TIRF microscopy reveals that adding ΔMod, V1ab4, and VASP to a reaction containing Arp2/3 and VCA suppresses filament branching and instead promotes the formation of unbranched actin bundles, similar to what is observed in the absence of Arp2/3 (Fig. 6C and movie S9).

We next asked how the actomyosin-dependent α-catenin-vinculin-VASP machinery affects Arp2/3-branched actin networks. Myosin alone deforms and disassembles Arp2/3-branched actin networks induced by micropatterned N-WASP (Fig. 6D and movie S10). With V1ab4 and VASP, Arp2/3-branched networks do not form on surfaces coated with both N-WASP and α-catenin, and VASP is not recruited to α-catenin–coated micropatterns (Fig. 6E, left, and movie S10). Adding myosin to these conditions, however, drives strong VASP recruitment to α-catenin–coated micropatterns and leads to the growth of large, dynamic actomyosin bundles (Fig. 6E, right, and movie S10). The kymographs and distributions of actin fluorescence intensity along the regions decorated with α-catenin confirm that the α-catenin-vinculin-VASP machinery blocks Arp2/3 branching and, when activated by myosin, promotes actin assembly and bundle formation (Fig. 6, F and G).

To test whether the α-catenin-vinculin-VASP machinery can reorganize a preformed branched actin network, we designed an experiment in which preincubated α-catenin ΔMod, vinculin V1ab4, Atto 488-VASP, Alexa Fluor 647-actin, and profilin were added to a micropatterned surface precoated with N-WASP that had already initiated Arp2/3-mediated filament branching. Our observations indicate that the Arp2/3-induced actin network at the center of the N-WASP–coated patterned area does not undergo significant reorganization into bundles following the addition of ΔMod, V1ab4, and VASP. This is supported by the homogeneous actin fluorescence (fig. S10, A and B), the absence of filament coalescence into compact structures on the kymographs (fig. S10C), and the fluorescence profiles measured at different time points (fig. S10D). Atto 488-VASP, added with ΔMod and V1ab4, does not penetrate the central network but instead accumulates at the periphery, where it associates with the growth of dense actin filament bundles (fig. S10, A to C). Quantification of the outside/inside fluorescence ratios for VASP and actin confirms that VASP associates only weakly with the central branched network compared with the peripheral bundles (fig. S10E). Together, these results reveal a principle of mutual exclusion, whereby α-catenin, vinculin, and VASP inhibit the initiation of Arp2/3-mediated branched networks but cannot remodel them once established, thereby ensuring the spatial and temporal segregation of branched and bundled actin assemblies.

## DISCUSSION

**Fig. 7.**
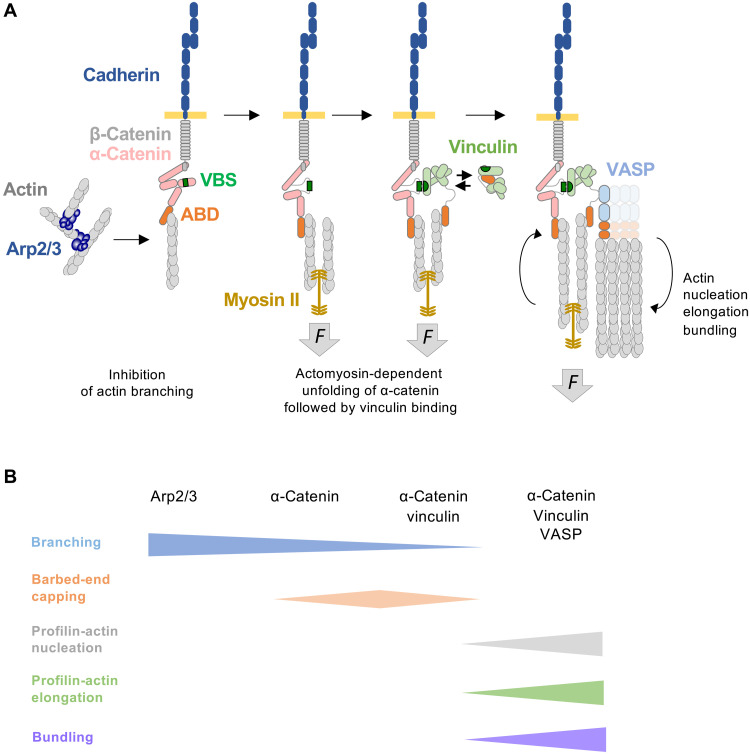
Model depicting the actomyosin-dependent assembly of α-catenin, vinculin, and VASP, which coordinates actin polymerization and organization at AJs. (**A**) Schematic of the gradual assembly of the AJ proteins in response to actomyosin-generated force (*F*), leading to the stimulation of actin filament nucleation, elongation, and bundling. (**B**) Progression of actin-regulatory activities by the indicated proteins as revealed in this study. The progression of activities shown in (B) corresponds to the sequential protein assembly illustrated in (A).

Our study demonstrates that α-catenin, vinculin, and VASP assemble in response to actomyosin contractile forces. The resulting machinery suppresses Arp2/3-mediated actin branching while promoting the nucleation and elongation of linear actin filaments that subsequently organize into bundles (Fig. 7).

Isolated proteins have often been considered as functional entities, and many studies have naturally sought to correlate their actin polymerization activities measured in vitro with actin dynamics observed in cells. In reality, many proteins are only parts of more complex machineries, as our study illustrates. α-Catenin is an inhibitor of actin filament barbed-end elongation (*43*), while vinculin caps and nucleates filaments (*33*), and VASP combines capping, nucleation, and elongation activities (*37*–*40*). Unexpectedly, we show here that the activity that results from their combination is not the sum of their individual activities. However, this process appears to be dependent on VASP’s activities, particularly nucleation and elongation, which are modulated by vinculin and α-catenin.

The mechanism of actin nucleation by VASP is not fully understood, but we hypothesize that its tetrameric state enables the actin-binding domains to bring actin monomers into proximity, facilitating the formation of an actin filament nucleus (*40*, *48*–*50*). It is possible that in our in vitro assays, the formation of a complex containing α-catenin dimers, vinculin dimers, and VASP tetramers results in complexes containing up to four VASP tetramers, further increasing the efficiency of actin nucleation. In cells, however, β-catenin prevents α-catenin homodimerization (*17*), but actin binding to α-catenin induces the oligomerization of the cadherin-catenin complex (*51*). Vinculin homo-oligomer formation could also occur at AJs and increase the nucleation activity of the α-catenin-vinculin-VASP machinery (*52*). This nucleation mechanism likely operates in cells, as we consistently observed it across varying ionic strengths and in the presence of profilin. The multiple oligomerization mechanisms associated with the α-catenin-vinculin-VASP machinery largely explain the actin filament bundling activity we observed.

The mechanism of actin filament barbed-end elongation by VASP is well established and involves clustering of VASP tetramers, which allows multiple monomeric actin-binding domains to deliver actin monomers to barbed ends held by ABDs (*37*, *38*, *53*). The increased elongation observed in our assays is likely due to the clustering of VASP tetramers within α-catenin-vinculin-VASP complexes. In this context, VASP seems to protect the growing barbed ends from the capping activities of vinculin or α-catenin, as it protects against the action of the heterodimeric CP (*38*, *39*, *54*). However, our results cannot be explained by simple competition for barbed-end binding, as vinculin and α-catenin significantly enhance the activity of VASP. Notably, this stimulatory effect of the bundling proteins vinculin and α-catenin on VASP resembles the effect of fascin on the *Drosophila* Ena protein, a member of the Ena/VASP family (*55*), suggesting a general mechanism by which bundling proteins promote VASP-mediated elongation.

We showed that the α-catenin-vinculin-VASP machinery assembles in an actomyosin-dependent manner, triggered by mechanical exposure of α-catenin’s VBS. Several studies suggest that this response is amplified at multiple levels. First, both α-catenin-actin and vinculin-actin interactions form catch bonds that strengthen under force (*27*, *30*, *56*). In addition, the slow dissociation of vinculin from the VBS locks α-catenin in an extended conformation (*25*). Single-molecule assays also revealed that vinculin forms a force-dependent catch bond with actin filaments, with longer bond lifetimes when force is applied toward the pointed end compared to the barbed end (*56*, *57*).

Our biochemical findings must be considered in the context of AJs. In cells, AJ formation involves actin polymerization, which has been proposed to promote lateral clustering of cadherins (*58*), while actomyosin-generated tension reinforces the junctions (*24*). In Madin-Darby canine kidney cells, cadherin puncta observed in fluorescence microscopy correspond to interdigitations of the plasma membrane (*10*). These interdigitations are membrane protrusions whose formation depends on actin polymerization driven by the Arp2/3 complex, the Ena-VASP-like protein, and the collapsin response mediator protein–1 (*59*). Electron microscopy experiments revealed two distinct actin networks associated with AJs, a branched actin network adjacent to the junctional plasma membrane and a bundle of filaments parallel to the membrane, positioned away from the cadherin-rich zone (*60*). Junctional myosin isoforms also display specific localizations and roles. NMIIA associates with actin bundles to generate force, while NMIIB binds to the branched network to promote force transmission (*12*). Our findings indicate that the application of actomyosin forces to α-catenin, which in turn recruits vinculin and VASP, suppresses Arp2/3-mediated branching and promotes the formation of linear bundles. Supporting this, NMIIB knockdown cells display AJs with less open α-catenin and thicker Arp2/3-containing actin networks (*12*).

In addition to Arp2/3 and α-catenin-vinculin-VASP machineries, formins, such as Diaphanous-1 and formin-1, known to promote the elongation of unbranched actin filaments (*42*), play a role in actin assembly at AJs (*61*–*63*). Further studies are needed to understand the organization and dynamics of actin networks that result from coordination between the activities of Arp2/3, formins, α-catenin-vinculin-VASP, and the myosins NMIIA and NMIIB.

## MATERIALS AND METHODS

### cDNA constructs

The cDNAs encoding mouse α-catenin WT and ΔMod were cloned into the pDW363 plasmid with a C-terminal His tag using the cDNAs previously reported (*22*, *64*). cDNA encoding human vinculin E28K/D33H/D110H/R113E/N773I/E775K (V1ab4) was synthesized and subcloned into the Nco I site of pET-3d by Genscript, with a C-terminal His tag as previously described (*41*). The cDNA encoding human vinculin 1-851, corresponding to Vh, was cloned into a pGEX-6P1-eGFP plasmid as previously described (*65*). The cDNAs encoding full-length human N-WASP cloned into pFastBacHTa and its VCA domain cloned into pGEX4T-1 have been described earlier (*66*). The DNA encoding human profilin I was described previously (*67*). The cDNAs encoding the α1 and β2 subunits of the mouse CP were cloned into pRSFDuet-1 as previously described (*67*). The cDNA encoding human full-length VASP was cloned into a pGEX-6P1 plasmid (gift from G. Scita, IFOM, University of Milan, Italy).

### Protein purification

Rabbit skeletal muscle actin was purified from acetone powder (*68*). Briefly, cycles of polymerization and depolymerization were followed by dialysis in 5 mM tris, pH 7.8, 0.2 mM adenosine 5′-triphosphate (ATP), 0.1 mM CaCl_2_, 0.01% NaN_3_, and 1 mM dithiothreitol (DTT) and gel filtration on a Superdex G-200 column (GE Healthcare). Actin was labeled with *N*-pyrenyliodoacetamide and Alexa Fluor 488, Alexa Fluor 647N (Invitrogen), or Atto 488 (Atto-TEC) succinimidyl ester as previously described (*65*, *69*). Myosin II was extracted from rabbit skeletal muscles in a buffer containing 500 mM KCl and 100 mM K_2_HPO_4_ (*70*). Briefly, after grinding and centrifugation, the actin-containing pellet was discarded. The supernatant underwent cycles of precipitation in low-salt buffer, followed by centrifugation and resuspension in high-salt buffer. Last, myosin II was dialyzed in 20 mM KH_2_PO_4_/K_2_HPO_4_, pH 7.5, 500 mM KCl, and 1 mM EDTA and stored at −20°C after addition of 50% glycerol.

All recombinant proteins in this study were expressed using the same protocol. After plasmid transformation in *Escherichia coli* (BL21 DE3, Invitrogen), bacteria were grown in 3 to 6 liters of LB medium containing appropriate antibiotics (ampicillin or kanamycin, 0.1 mg ml^−1^) at 37°C until the absorbance at 600 nm reached 0.6 to 0.8. Isopropyl 1-thio-β-d-galactopyranoside (1 mM) was added to the medium to induce the expression of the recombinant proteins of interest during 16 hours at 16°C. After centrifugation, the α-catenin WT, α-catenin ΔMod, and VASP FL bacterial pellets were lysed by sonication in 20 mM tris, pH 7.8, 500 mM NaCl, 1 mM β-mercaptoethanol, benzamidine (10 μg ml^−1^), and 1 mM phenylmethylsulfonyl fluoride. The V1ab4 bacterial pellets were lysed by sonication in 20 mM tris, pH 8.0, 1 M NaCl, 1 mM β-mercaptoethanol, benzamidine (10 μg ml^−1^), and 1 mM phenylmethylsulfonyl fluoride.

α-Catenin WT and ΔMod were purified as previously described (*64*). Briefly, lysates of His-tagged proteins were bound to Ni-NTA (Ni^2+^-nitrilotriacetic acid)-sepharose affinity chromatography (Qiagen); washed with 50 mM tris, pH 7.8, 500 mM NaCl, 20 mM imidazole, and 1 mM β-mercaptoethanol; eluted with 50 mM tris, pH 7.8, 500 mM NaCl, 250 mM imidazole, and 1 mM β-mercaptoethanol; and purified on a gel filtration column (Superdex 200, 16/60, GE Healthcare). α-Catenin constructs were finally dialyzed in 20 mM tris, pH 7.8, 150 mM KCl, and 1 mM DTT; frozen in liquid nitrogen; and stored at −80°C. V1ab4 was purified by Ni-NTA-sepharose affinity chromatography (Qiagen), followed by a Q-Sepharose ion exchange column. V1ab4 protein was finally dialyzed in 20 mM tris, pH 7.8, and 1 mM DTT; frozen in liquid nitrogen; and stored at −80°C.

Human full-length VASP lysates were first bound to glutathione sepharose in 50 mM tris, pH 7.8, and 500 mM NaCl and then cleaved from glutathione *S*-transferase by PreScission protease. The protein was dialyzed in 20 mM tris, 100 mM KCl, and 1 mM DTT; frozen in liquid nitrogen; and stored at −80°C.

Well-established protocols were used to purify Vh-eGFP (*65*), bovine brain Arp2/3 (*71*), spectrin-actin seeds (*72*), human recombinant profilin I and mouse CP (*67*), and human full-length N-WASP and its VCA domain (*66*). For microscopy experiments, we use proteins that are either nonlabeled or labeled with Alexa Fluor 568-maleimide (Invitrogen) or Atto 488-maleimide (ATTO-TEC) as previously described (*41*).

### Observation of micropatterned surfaces using TIRF microscopy

The assay used to observe the assembly of α-catenin, vinculin, VASP, actin, and myosin on micropatterned surfaces was inspired by existing protocols (*44*, *65*, *73*, *74*), with the following modifications. Glass coverslips (24 mm by 40 mm, no. 1.5, Thermo Fisher Scientific/Menzel-Glaser) were first washed with Milli-Q water and ethanol, sonicated, and irradiated for 1 min under a deep ultraviolet (UV) lamp (Ossila). The coverslips were incubated for 2 hours in poly(l-lysine)-*g*-poly(ethylene glycol) (PLL-*g*-PEG; 0.1 mg ml^−1^) (SuSoS) dissolved in 10 mM Hepes, pH 7.8, and washed with Milli-Q water. The chrome-quartz photomask (Toppan, France), designed with hexagon of 20-μm edges and 4-μm thickness (Fig. 1A), was cleaned by deep UV irradiation for 1 min, placed on the PLL-*g*-PEG–coated coverslip, and exposed to deep UV for 5 min. The chamber was made of a micropatterned coverslip attached to a glass slide (Super Frost, Thermo Fisher Scientific) with double-sided adhesive tape. The volume of a chamber was 100 μl. Two different setups were used: one to investigate the mechanosensitive assembly of the proteins and the other to examine their actin polymerization activity.

To study the mechanosensitive formation of the α-catenin-vinculin-VASP machinery, the chamber was first incubated with Alexa Fluor 568–labeled α-catenin WT (10 μM) for 5 min at room temperature. Unbound α-catenin was washed out with 300 μl of wash buffer [5 mM tris, pH 7.8, 200 μM ATP, 5 mM 1,4-diazabicyclo(2,2,2)-octane (DABCO), 1 mM MgCl_2_, 200 μM EGTA, 20 mM DTT, and 25 mM KCl]. The surface was passivated with 300 μl of wash buffer containing 1% BSA for 5 min at room temperature. Last, 100 μl of the reaction was added, and the chamber was sealed with VALAP (1:1:1 mixture of Vaseline, lanolin, and paraffin). A typical reaction contained 2.4 μM actin (containing 2% Alexa Fluor 647N–labeled actin), 10 μM profilin, 50 nM myosin II, 1% BSA, a salt mix (2 mM MgCl_2_, 0.2 mM EGTA, and 25 mM KCl), and an ATP regenerating mix [2 mM ATP, 2 mM MgCl_2_, 10 mM creatine phosphate, and creatine kinase (3.5 U/ml)] in G-fluo buffer (5 mM tris, pH 7.8, 200 μM ATP, 0.4% methylcellulose, 5 mM DABCO,1 mM MgCl_2_, 200 μM EGTA, and 2 mM DTT). Additional proteins such as Vh-eGFP, V1ab4 (nonlabeled or labeled with Alexa Fluor 568 or Atto 488), VASP-FL (nonlabeled or labeled with Atto 488), or Alexa Fluor 568–labeled myosin II were also added.

To study the polymerization activity of the α-catenin-vinculin-VASP machinery, we first preformed the α-catenin ΔMod/V1ab4/VASP complex by incubating the three proteins at 1.6 μM (1:1:1) for 30 min at room temperature. The chamber was then incubated with the three-protein mix for 5 min at room temperature. Unbound proteins were washed out using 100 μl of the actin-containing reaction mix, and the chamber was sealed with VALAP (1:1:1 mixture of Vaseline, lanolin, and paraffin). A typical reaction mix contained either 1, 2.4, or 5 μM actin (containing 2 or 10% Alexa Fluor 647N–labeled actin); 5, 10, or 20 μM profilin depending on the actin concentration used in the experiment; 1% BSA; and a salt mix (2 mM MgCl_2_, 0.2 mM EGTA, and 25 mM KCl) in G-fluo buffer (5 mM tris, pH 7.8, 200 μM ATP, 0.4% methylcellulose, 5 mM DABCO, 1 mM MgCl_2_, 200 μM EGTA, and 2 mM DTT).

Micropatterned samples were imaged using TIRF microscopy on a Nikon Ti Eclipse E microscope equipped with a 60× oil immersion Apochromat objective [numerical aperture (NA), 1.49] and a Hamamatsu Orca Flash04 sCMOS camera. Fluorescent proteins were excited using 488-, 561-, and 642-nm lasers.

### FRAP experiments

Images were initially acquired at 10-s intervals during a 10-min prebleach period. Alexa Fluor 647N–labeled actin was then photobleached within rectangular regions of interest (ROIs) (100-by-200 pixels) using 30 consecutive 20-ms pulses of a 647-nm laser at 80% power. Postbleach recovery was monitored for 25 min, with images acquired every 10 s using the same laser.

Samples were imaged on a Nikon Ti Eclipse E inverted microscope equipped with a 60× oil immersion Apochromat objective (NA, 1.49) and a Hamamatsu Orca Flash04 sCMOS camera. Photobleaching was performed with the iLas 2 system (GATACA Systems).

### Actin polymerization assays

Actin polymerization was measured by the increase in fluorescence of 10% pyrene-labeled actin. For barbed-end elongation measurements, 100 pM spectrin-actin seeds were added to the reaction, and polymerization was induced by adding 100 mM KCl, 1 mM MgCl_2_, and 0.2 mM EGTA to a solution of 10% pyrene-labeled Ca-ATP-G-actin containing the proteins of interest. To test the nucleation activity of proteins, spontaneous polymerization was induced by adding 25 mM KCl, 1 mM MgCl_2_, and 0.2 mM EGTA to a solution of 10% pyrene-labeled Ca-ATP-G-actin in the presence of the proteins of interest. Fluorescence measurements were carried out using a Safas Xenius FLX spectrofluorometer (Safas, Monaco). The graphs and plots were generated with Excel or KaleidaGraph. The experiments were repeated two to three times, consistently yielding the same conclusions. Kinetics were quantified by measuring either the maximum rate or the half-times of actin polymerization. The apparent affinities for actin barbed ends of V1ab4 and ΔMod were determined using the method previously described in (*33*).

### Light scattering measurements and microscopy observation of actin bundling

Actin filament bundling during polymerization was examined by measuring light scattering at 400 nm using a Safas Xenius FLX spectrophotometer (Safas, Monaco). Polymerization was induced by adding 25 mM KCl, 1 mM MgCl_2_, and 0.2 mM EGTA to a solution of 2% Alexa Fluor 488–labeled G-actin in the presence of the proteins of interest. A 5-μl sample was taken from the reaction after 1500 s for epifluorescence microscopy observation. The light scattering kinetics were analyzed and plotted using Excel or KaleidaGraph. For epifluorescence microscopy, images were acquired with an Olympus IX71 inverted microscope equipped with a 60× oil immersion objective (Olympus; NA, 1.45), coupled to an electron multiplying charge-coupled device camera (Cascade, Photometrics), and illuminated using an EXFO X-Cite Series 120 lamp with appropriate filter sets for the fluorophores used. Images were acquired with MicroManager and processed with ImageJ software. The experiments were repeated three times, consistently yielding the same conclusions.

### Observation of single actin filaments using TIRF microscopy

Our protocol is based on a modification of previously published methods (*33*, *75*). After sonication with Milli-Q water and ethanol, the coverslips (24 mm by 40 mm; Thermo Fisher Scientific/Menzel-Glaser) were irradiated for 1 min under a deep UV lamp (Ossila) and then incubated with PLL-*g*-PEG (0.1 mg ml^−1^; SuSoS) dissolved in 10 mM Hepes, pH 7.4, for 1 hour at room temperature. The coverslips were then washed with Milli-Q water and dried. A flow chamber, with a typical volume of 50 to 70 μl, was created by sticking the PLL-PEG–passivated side of the coverslip to a glass slide (Super Frost, Thermo Fisher Scientific) using a double-sided adhesive tape. After incubation with washing buffer (5 mM tris, pH 7.8, 200 μM ATP, 1 mM DTT, 1 mM MgCl_2_, and 25 or 100 mM KCl) for 1 min, the chamber was saturated with 10% BSA for 5 min and then washed with washing buffer. A reaction composed of 1.5 μM G-actin (10% Alexa Fluor 488– or Atto 488–labeled) in 5 mM tris, pH 7.8, 200 μM ATP, 0.4% methylcellulose, 5 mM DABCO, 100 or 25 mM KCl, 1 mM MgCl_2_, 200 μM EGTA, and 10 mM DTT supplemented with our proteins of interest was injected in the chamber sealed with oil or VALAP. Single filaments were observed using an Olympus IX71 inverted microscope equipped with a 60× oil immersion objective (Olympus; NA, 1.45); illuminated with a 488-nm, 50-mW laser (Errol); and coupled to an electron multiplying charge-coupled device camera (Cascade, Photometrics).

### Image analysis

All time-lapse videos and images were acquired using either MicroManager or MetaMorph software and subsequently analyzed with ImageJ (National Institutes of Health). For steady-state data (Fig. 1, B to E), each point on the dot plots represents the mean fluorescence intensity of a single edge of a hexagon (20 to 30 edges per frame; 10 frames per condition). The total actin intensity (Figs. 3 and 5) was calculated by summing the pixel values within a single frame and subtracting the mean background intensity from the control condition lacking actin (10 images per condition).

For FRAP experiments, fluorescence recovery after bleaching was calculated on defined structures within the bleached region (three structures per condition). To characterize the effect of our proteins of interest on individual actin filaments observed using TIRF microscopy, we quantified both filament density and elongation rates across conditions. The single filament density (Fig. 2) was determined by manually counting the number of filaments at successive time points (every 100 s). To measure filament elongation rates, time-lapse movies were first temporally calibrated on the basis of acquisition settings and corrected for sample drift using the ImageStabilizer plug-in in ImageJ. ROIs were defined using segmented line selections drawn along individual filaments, oriented from the pointed end toward the barbed end to follow the direction of polymerization. For each ROI, a kymograph was generated using the KymoToolBox plug-in in ImageJ, and the elongation rate was calculated by measuring the slope of the filament trace on the kymograph.

For the mechanosensitivity experiments (Fig. 4 to 6), multichannel image stacks were aligned using the Linear Stack Alignment plug-in in FIJI. Patches, defined as regions of high actin or myosin intensity, were manually selected on the actin or myosin channel using freehand ROI selection. Each ROI was then applied to the 488- or 561-nm channels (corresponding to Vh-eGFP or Alexa Fluor 568-V1ab4) to measure the corresponding maximum fluorescence intensity. The same ROI was subsequently repositioned to a nearby area lacking patches (regions of low actomyosin intensity), and the maximum intensity was measured again. Each patch was thus associated with a pair of values (high and low actin or myosin intensity) used for comparative analysis in the graph. The actin/VASP fluorescence ratio (Fig. 5) was calculated by measuring the mean fluorescence intensity of both actin and VASP within each ROI over a 1200-s period (1 frame every 10 s).

For correlation analysis of Vh with either myosin II or actin (Fig. 4D), ROIs were defined by drawing line selections along individual pattern edges in the 488-nm channel (Vh-eGFP). The same ROIs were then applied to the 561-nm channel (Atto 568-myosin II) and the 642-nm channel (Alexa Fluor 647-actin). For each channel, a plot profile was generated, yielding a dataset in which each point corresponded to three fluorescence intensity values (Vh, myosin II, and actin). Scatterplots were constructed to represent Vh fluorescence as a function of either myosin II or actin fluorescence, and Pearson correlation coefficients (*r*) were calculated using the Pearson function in Excel. The same procedure was used to assess the correlation of V1ab4 with actin (Fig. 4G).

To quantify the expansion of VASP patches, a straight line was drawn across the extremities of each patch in the final frame of the movie to generate a kymograph. A binary image was then generated in ImageJ using the MinError thresholding method, from which a plot profile was obtained. The patch width was measured in the final frame and converted to micrometers.

Plots were generated using either KaleidaGraph or GraphPad Prism (version 10.4.1). All experiments were independently repeated and yielded consistent results as indicated in the legends of the figures. Statistical analysis was performed using the Student’s *t* test with the parameters indicated in the legends of the figures.
