# Supplementary material for: Actomyosin-dependent assembly of the mechanosensitive machinery from adherens junctions triggers actin polymerization and organization
Source: Sci Adv. 2026 Jan 1;12(1):eady4863. doi: 10.1126/sciadv.ady4863 (PMC12757075; doi:10.1126/sciadv.ady4863)
Supplement: Supplementary file 1 — Figs. S1 to S10 Legends for movies S1 to S10 Legend for data file [file sciadv.ady4863_sm.pdf]

Science Advances
AAAS

Supplementary Materials for

Actomyosin-dependent assembly of the mechanosensitive machinery from adherens junctions triggers actin polymerization and organization

Aurélie Favarin *et al.*

Corresponding author: Christophe Le Clainche, christophe.leclainche@i2bc.paris-saclay.fr

Sci. Adv. **12**, eady4863 (2026)
DOI: 10.1126/sciadv.ady4863

The PDF file includes:

Figs. S1 to S10
Legends for movies S1 to S10
Legend for data file

Other Supplementary Material for this manuscript includes the following:

Movies S1 to S10
Data file

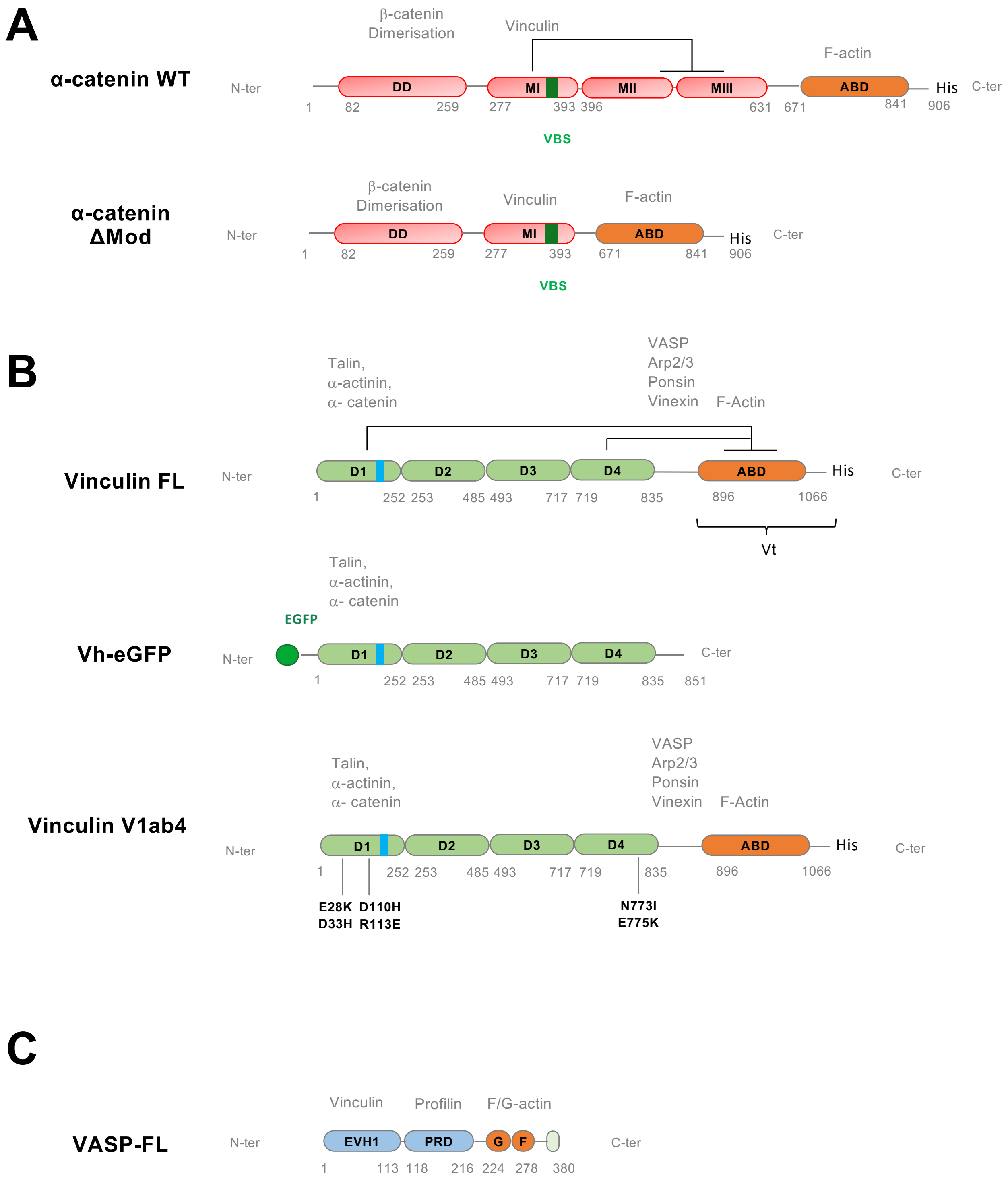

Supplementary Figure 1. Schematic representation of protein constructs used in this study. (A) α-catenin WT and ΔMod: DD, dimerization domain; MI-MII-MIII, modulation domains; VBS, vinculin binding site (green). **(B)** Vinculin Vh-eGFP, V1ab4: Vt, vinculin tail, α-catenin binding sites (blue); E28K/D33H, D110H/R113E, N773I/E775K, point mutations in V1ab4. **(C)** VASP FL: EVH1, Ena/VASP homology domain 1; PRD, proline-rich domain. **(A-C)** ABD, actin-binding domain (orange); N-ter, N-terminal; C-ter, C-terminal; proteins partners shown in grey bind to the corresponding domain of each protein of interest.

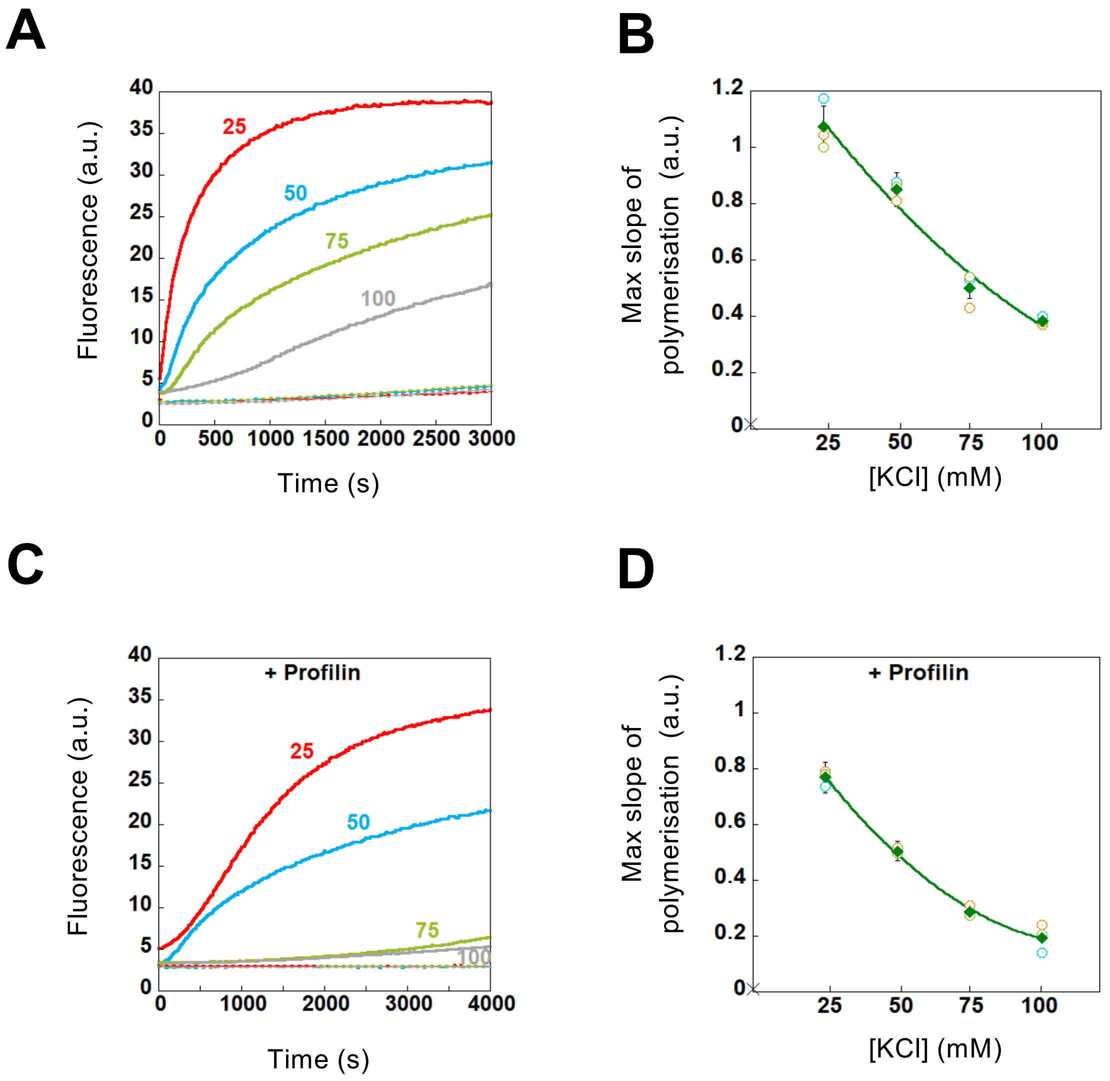

Supplementary Figure 2. Effect of profilin and ionic strength on the stimulation of actin polymerisation by α-catenin, vinculin and VASP. (A) Kinetics of spontaneous actin polymerisation (1.5 µM G-actin, 10% pyrene-labelled) measured in the presence of increasing concentrations of KCl, along with 1.5 µM ΔMod, 2 µM V1ab4, and 1 µM VASP. **(B)** Maximal slope of actin polymerisation under the conditions shown in (A). **(C)** Same experiment as in (A), performed in the presence of 10 µM profilin. **(D)** Maximal slope of actin polymerisation under the conditions shown in (C). **(A, C)** Dashed lines indicate control conditions with actin alone (A) or actin with profilin (C) at 25, 50, 70, and 100 mM KCl. (B, D) Open symbols in different colours represent three independent experiments under identical conditions; closed symbols indicate the mean of the triplicates. Data are presented as mean ± SEM (N = 3).

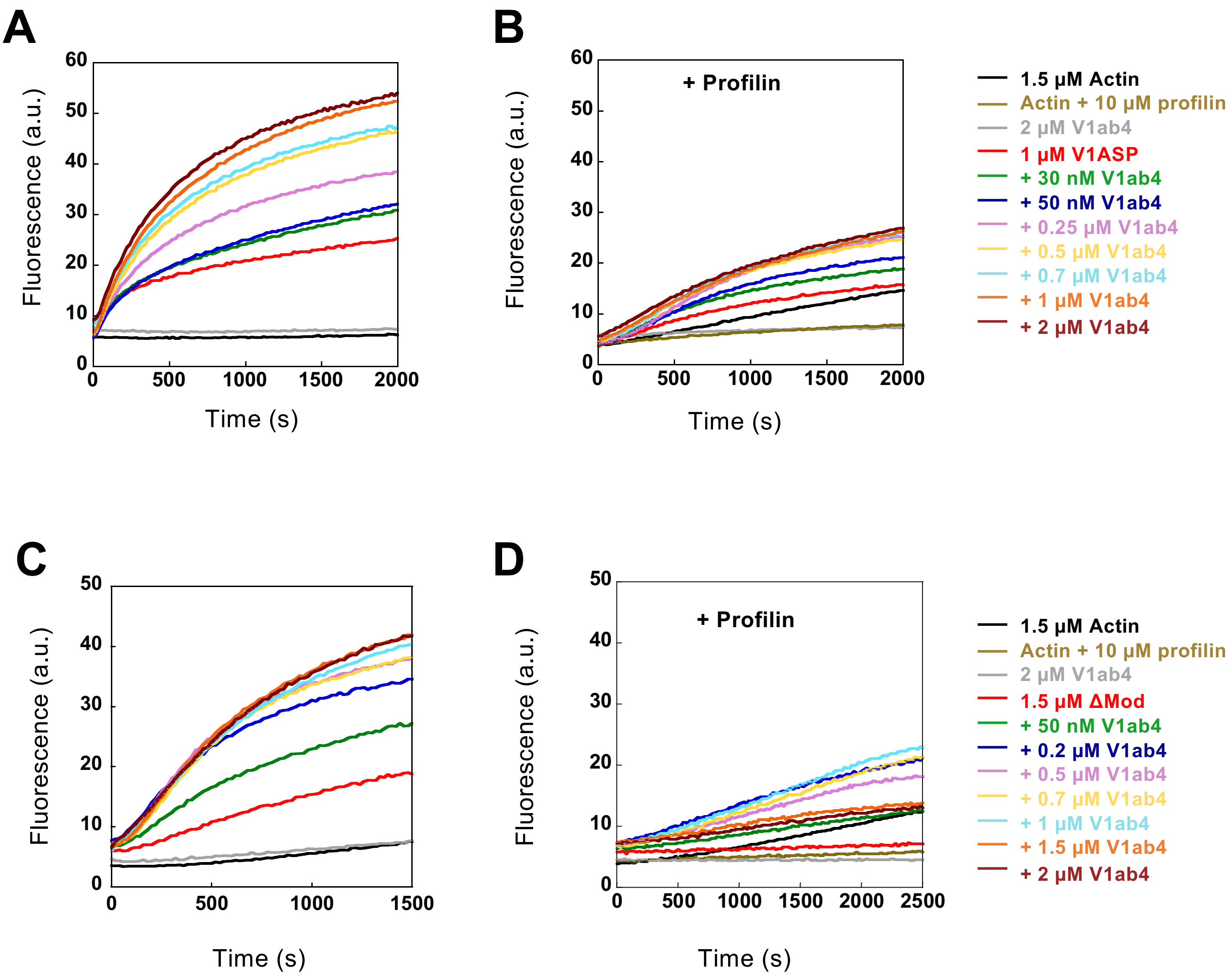

Supplementary Figure 3. V1ab4 cooperates with VASP and ΔMod to nucleate actin filaments.
(A-B) Spontaneous actin polymerisation (1.5 µM G-actin, 10% pyrene-labeled) was measured in the presence of increasing concentrations of V1ab4 in the presence of 1 µM VASP, and in the absence (A) or presence (B) of 10 µM profilin. **(C-D)** Spontaneous actin polymerisation (1.5 µM G-actin, 10% pyrene-labeled) was measured in the presence of increasing concentrations of V1ab4 in the presence of 1.5 µM ΔMod, and in the absence (C) or presence (D) of 10 µM profilin. All experiments where performed three times with the same results.

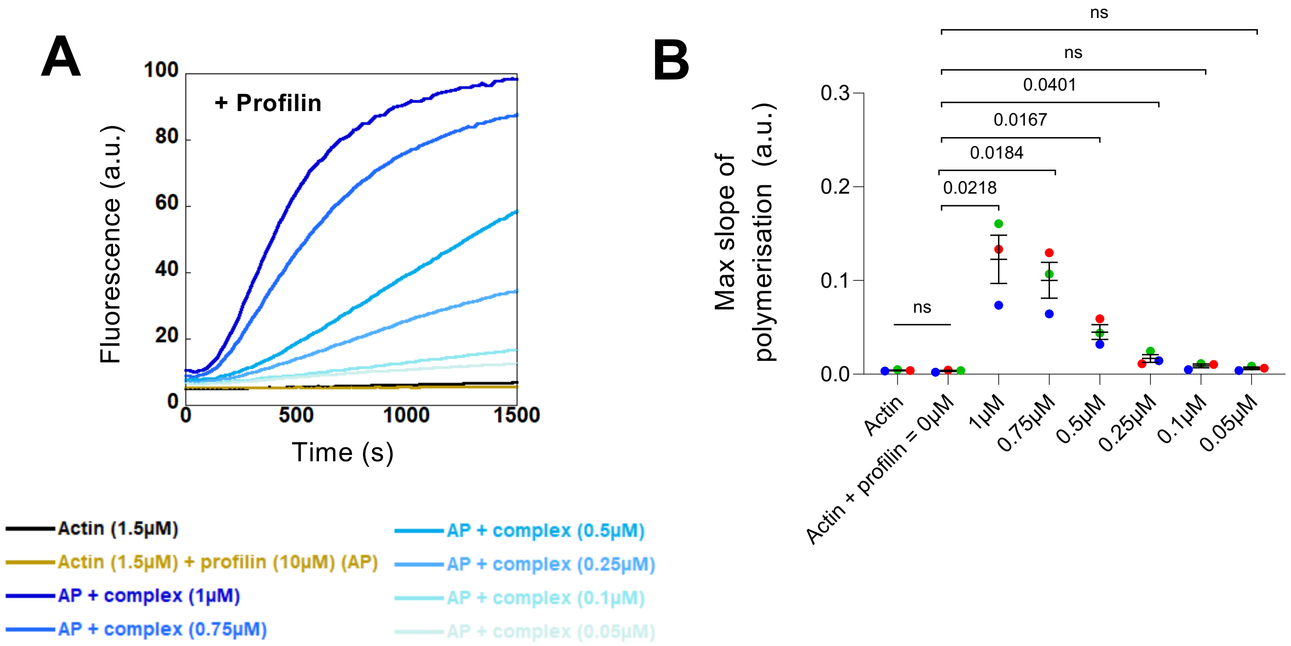

Supplementary Figure 4. Dose-dependent effect of the α-catenin-vinculin-VASP machinery on actin assembly. (A) Kinetics of spontaneous actin polymerisation (1.5 µM G-actin, 10% pyrene-labelled) were measured in the presence of decreasing concentrations of Δmod-V1ab4-VASP (complex), with AP (actin–profilin) and complex (x µM = x µM of each of the three proteins), at 25 mM KCl. **(B)** Maximal slope of actin polymerisation under the conditions shown in (A). Symbols of different colours represent three independent experiments under identical conditions. Data are presented as mean ± SEM (N = 3). p-values were calculated using a one-tailed unpaired t-test comparing group means.

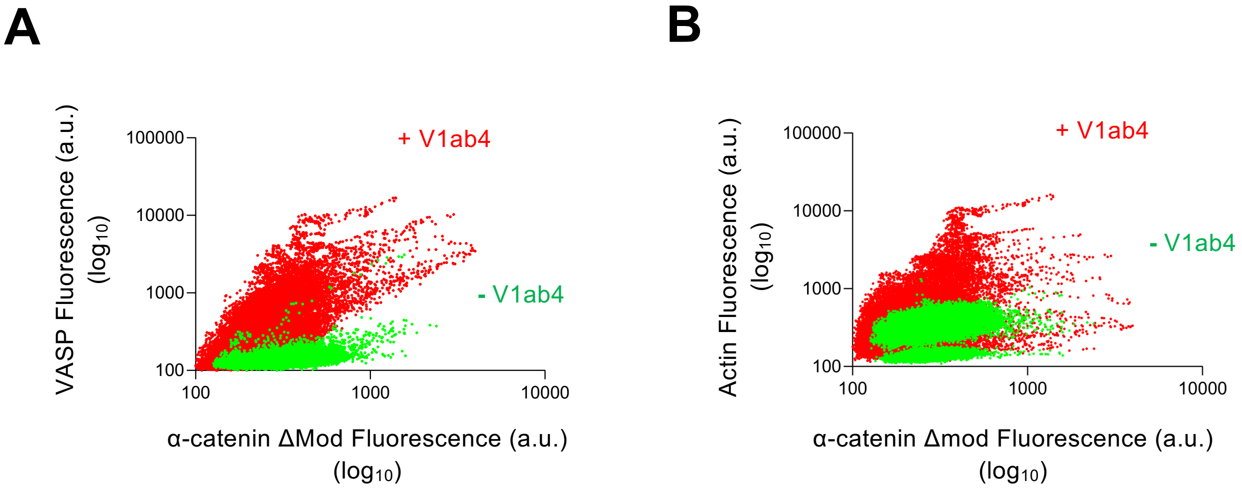

Supplementary Figure 5. Actin assembly depends on the recruitment of VASP to α-catenin ΔMod by V1ab4. (A–B) Quantifications of the experiment shown in Figure 2G. **(A)** VASP fluorescence intensity as a function of α-catenin ΔMod fluorescence intensity in micropatterns. **(B)** Actin fluorescence intensity as a function of α-catenin ΔMod fluorescence intensity in micropatterns. Each data point represents one pixel from n = 50–60 hexagon edges per condition, N = 3.

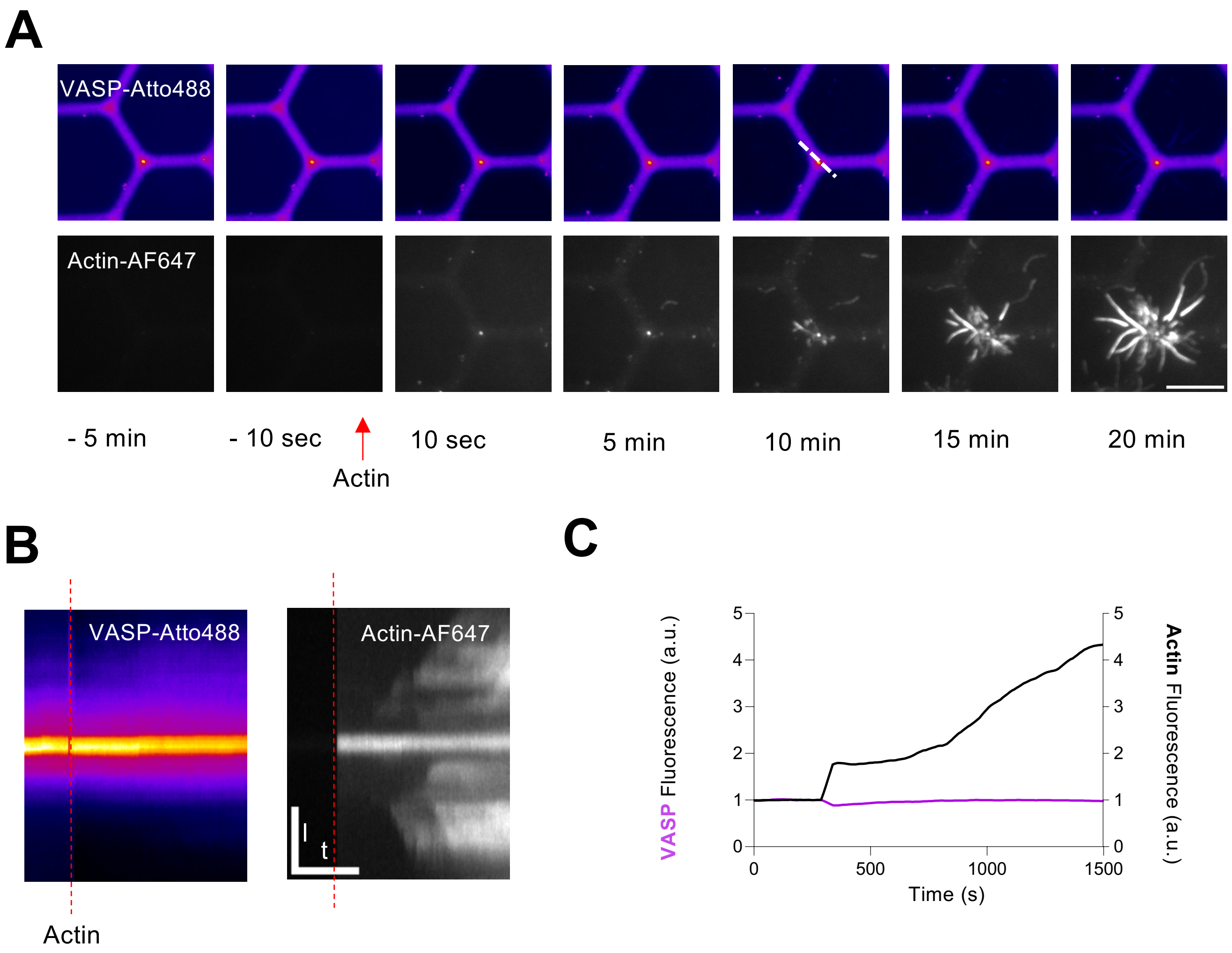

Supplementary Figure 6. Patch formation occurs independently of actin polymerisation and subsequently promotes actin assembly. (A) Time-lapse images of the assembly of 1 µM actin (2% AlexaFluor647N-labelled) in the presence of 5 µM profilin on a micropatterned surface pre-incubated with a mix of Δmod-V1ab4-VASP-Atto488 at 1.6 µM (1:1:1). VASP is shown using LUT Fire (ImageJ) and actin in grey. The arrow indicates the time of actin addition. Images were acquired using TIRF microscopy. Scale bar = 20 µm. **(B)** Kymographs corresponding to the region shown in (A). The red dashed line marks the time point at which actin was added. Scale bars: vertical (l) = 5 µm; horizontal (t) = 500 s. **(C)** Plot profile of fluorescence intensity over time of the aster. VASP (purple) and actin (black). This experiment was reproduced with the same results (N=2).

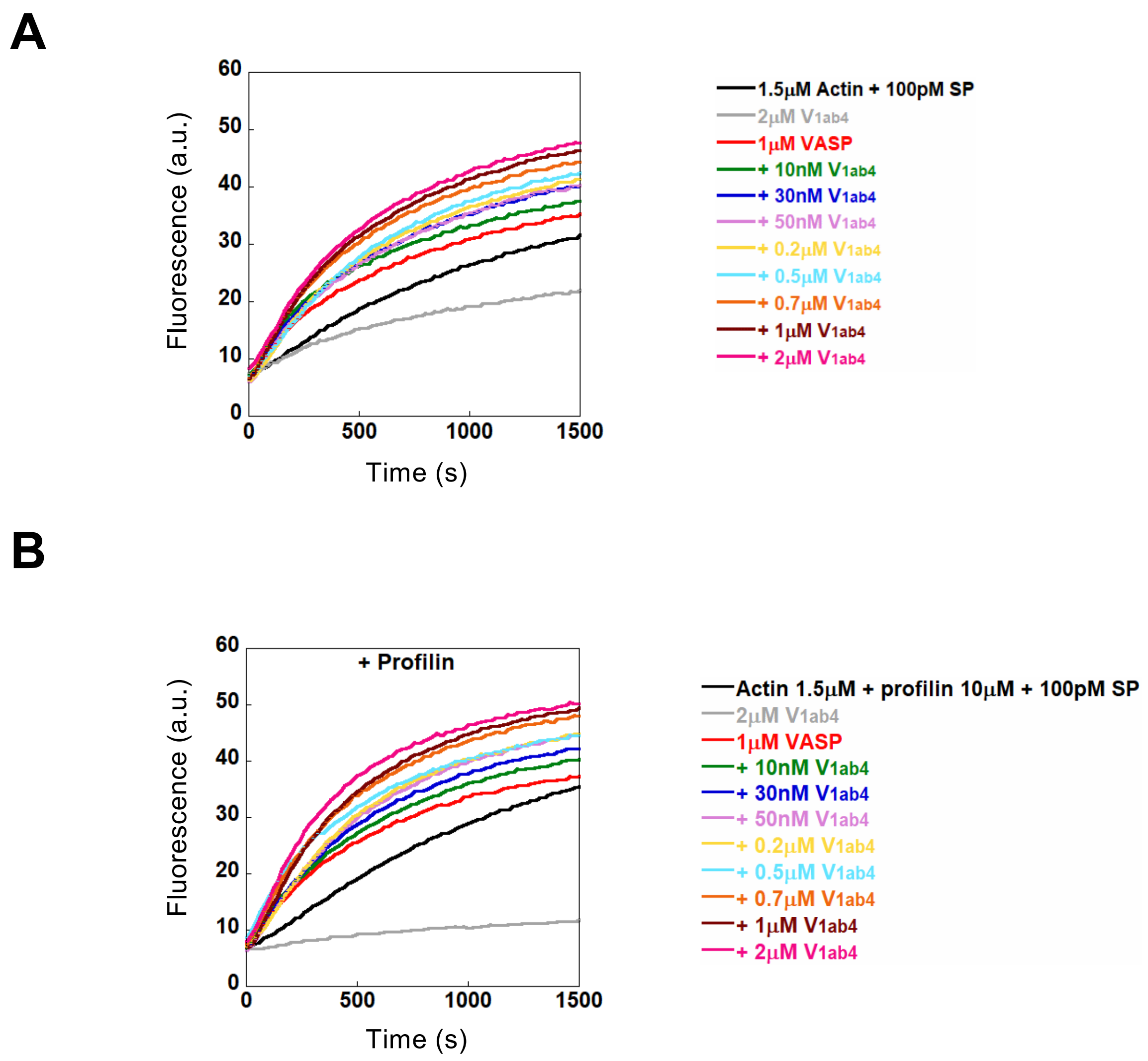

Supplementary Figure 7. V1ab4 cooperates with VASP to elongate actin filaments in the presence of profilin. (A, B) The elongation of actin filament barbed ends was measured in the presence of 100 pM spectrin–actin seeds (SP), 1.5 μM G-actin (10% pyrene-labelled), 1 μM VASP, and increasing concentrations of V1ab4, either in the absence (A) or presence (B) of 10 μM profilin. All experiments were independently replicated three times, yielding consistent results.

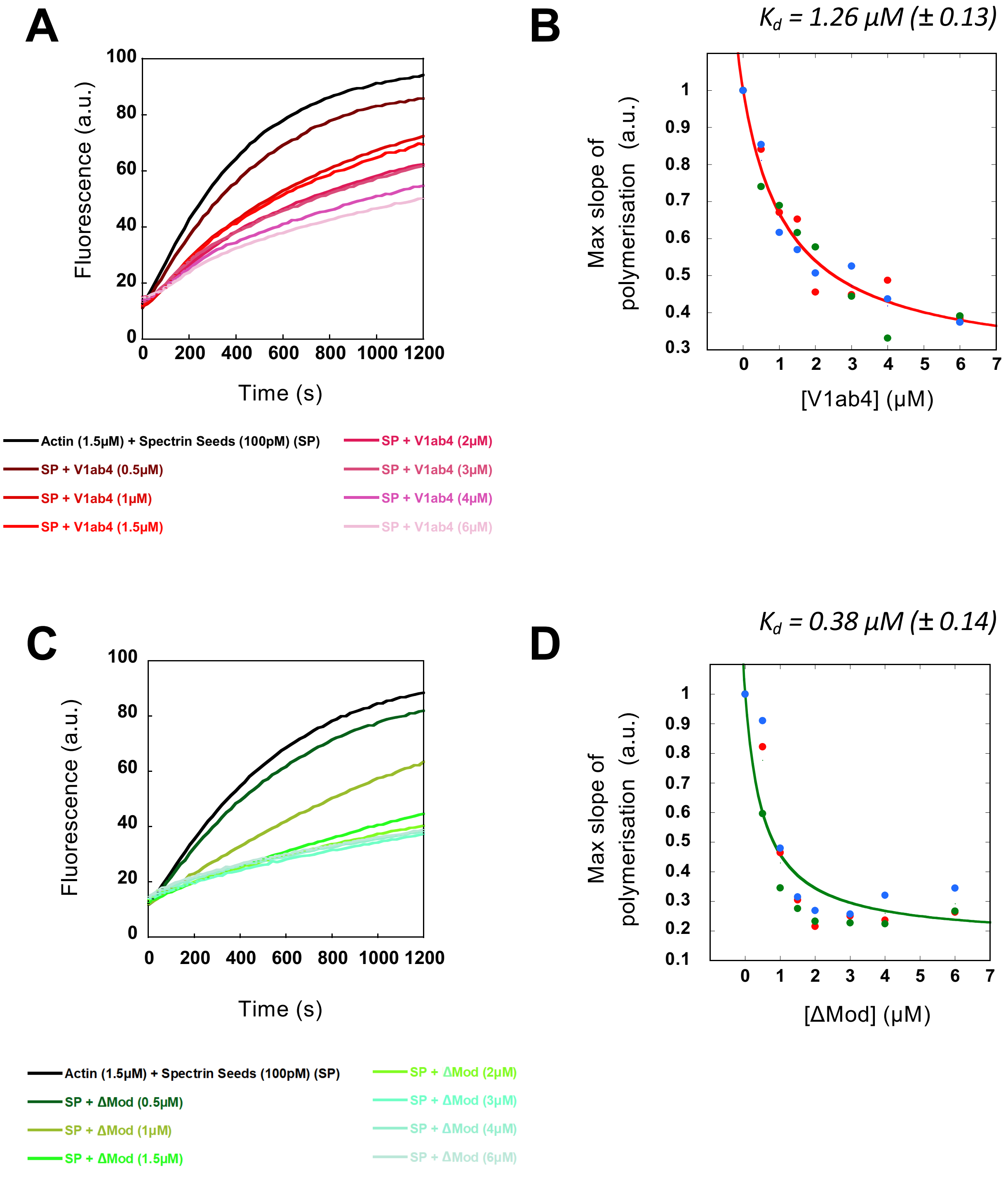

Supplementary Figure 8. V1ab4 and ΔMod binding affinity to actin filament barbed ends. (A, C) Barbed end elongation of 1.5 µM G-actin (10% pyrene-labelled) from 100 pM spectrin–actin seeds measured in the presence of increasing concentrations of V1ab4 (A) or α-catenin ΔMod (C) in high-salt buffer (100 mM KCl). **(B, D)** Maximal slope of barbed end elongation of 1.5 µM G-actin (10% pyrene-labelled) from 100 pM spectrin–actin seeds plotted as a function of increasing concentrations of V1ab4 (B) or α-catenin ΔMod (D) in high-salt buffer (100 mM KCl). Curve fit line is drawn based on the mean of three independent experiments (see Materials and Methods). Symbols of different colours represent three independent experiments performed under identical conditions.

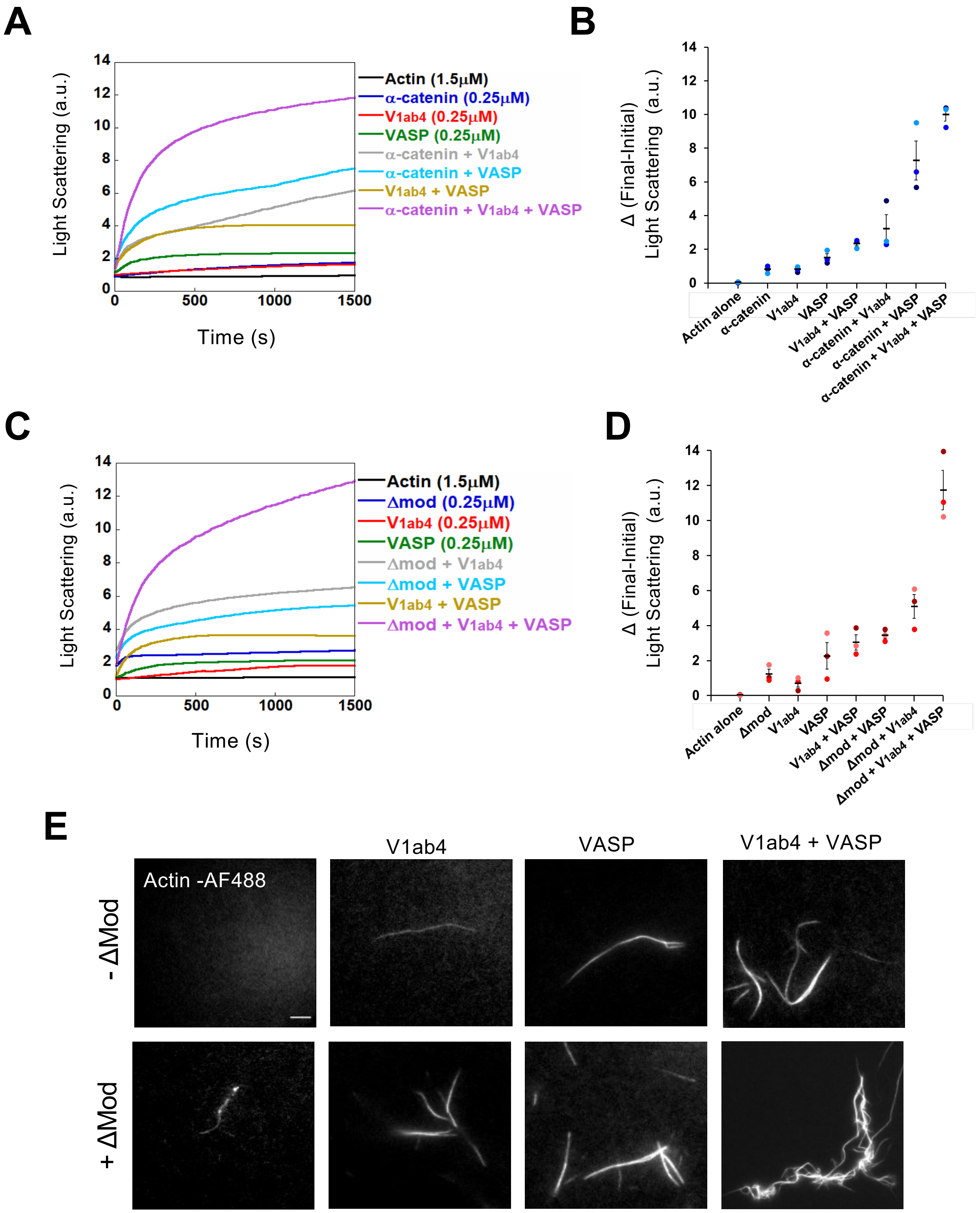

Supplementary Figure 9. α-catenin, vinculin and VASP bundle actin filaments synergistically. (A) Light scattering was measured during the polymerisation of actin alone (1.5 μM G-actin), and in the presence of all the combinations of 0.25 μM α-catenin WT, 0.25 μM V1ab4 and 0.25 μM VASP, in low salt (25 mM KCl). **(B)** Quantification of light scattering amplitude at 1500 s for each condition indicated in (A). **(C)** Light scattering was measured during the polymerisation of actin alone (1.5 μM G-actin), and in the presence of all the combinations of 0.25 μM α-catenin ΔMod, 0.25 μM V1ab4 and 0.25 μM VASP, in low salt (25 mM KCl). **(D)** Quantification of light scattering amplitude at 1500 s for each condition indicated in (C). **(A-D)** These experiments were performed three times in the same conditions. (B, D) Symbols with different colours represent three independent experiments. Error bars represent ± SEM (N=3). **(E)** Representative epifluorescence images of AlexaFluor488-labelled actin filaments observed alone and in the presence of all the combinations of ΔMod, V1ab4 and VASP. Conditions: 1.5 μM G-actin (2% AlexaFluor488-labelled), 0.25 μM Δmod, 0.25 μM V_{1ab4} and 0.25 μM VASP. Scale bar = 15 μm.

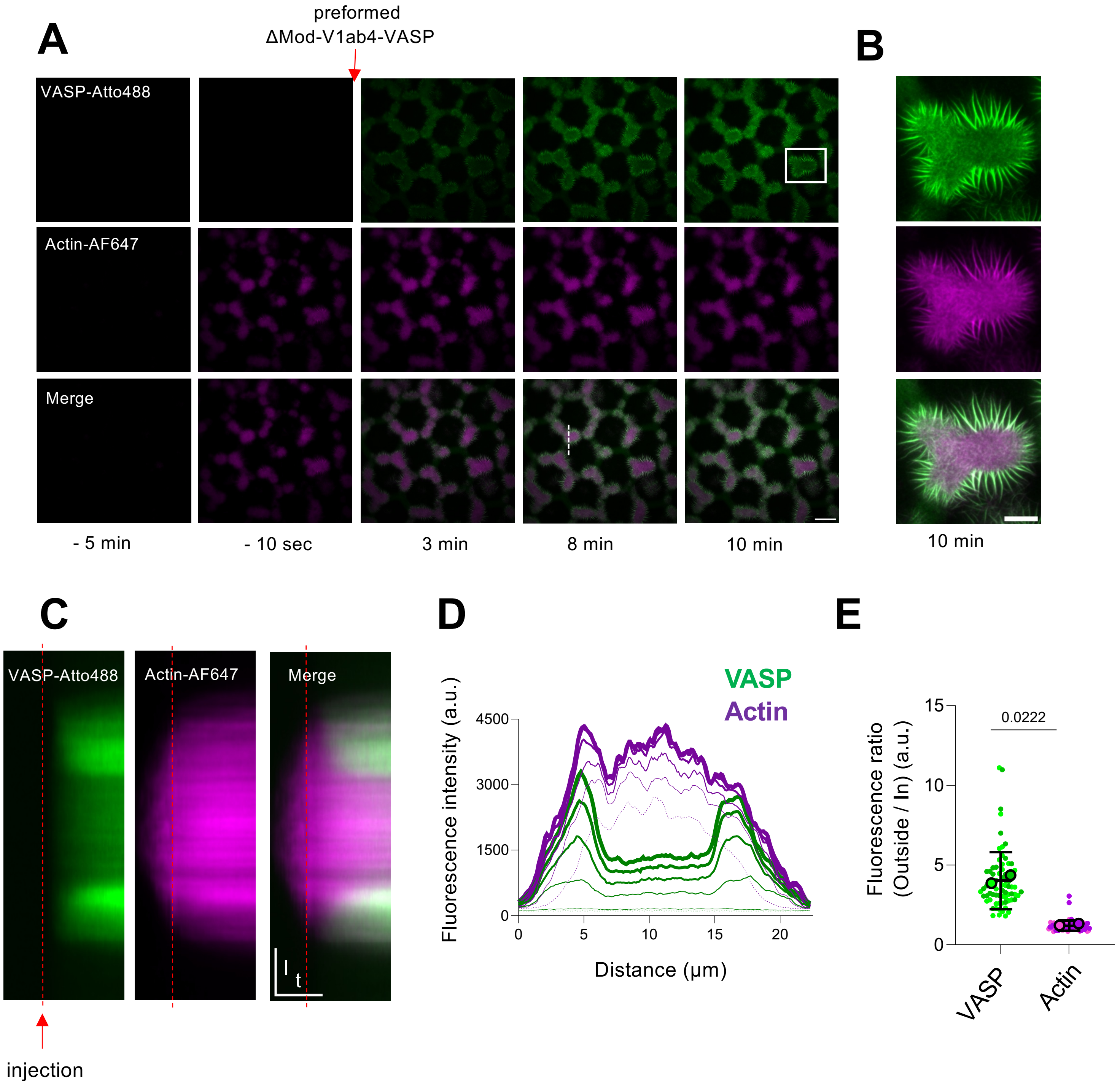

Supplementary Figure 10. α-catenin–vinculin–VASP does not remodel a pre-existing Arp2/3-mediated branched actin network. (A) Time-lapse images of the assembly of 1 µM actin (2% AlexaFluor647N-labelled) in the presence of 5 µM profilin and 50 nM Arp2/3 on a micropatterned surface pre-incubated with 50 nM N-WASP, before and after injection of preformed ΔMod–V1ab4–VASP at 0.5 µM (1:1:1), supplemented with 0.2 µM actin (2% AlexaFluor647N-labelled) and 1 µM profilin (to compensate for actin dilution induced by injection). VASP is shown in green and actin in magenta. Images were acquired using TIRF microscopy. Scale bar = 20 µm. **(B)** Magnified view of the boxed region in (A). Scale bar = 10 µm. **(C)** Kymograph showing VASP-Atto488 recruitment to the branched actin network following injection of the three proteins (red dashed line), along the dashed line indicated in (A). Scale bars: vertical (l) = 5 µm; horizontal (t) = 500 s. **(D)** Plot profile along the dashed line shown in (A) at different time points. The dashed trace shows VASP (green) and actin (magenta) intensities before injection of the three proteins; the trace width then increases over time (1.5, 3.5, 4.5, 6, and 8 min after injection). **(E)** Quantification of outside (linear bundles) versus inside (branched network) fluorescence ratios for VASP and actin. Symbols of different colours represent independent experiments performed under identical conditions. Each point corresponds to a fluorescence ratio between adjacent regions inside and outside of VASP-actin structures. Data are presented as mean ± SEM (N = 2). The p-value was calculated using a one-tailed paired t-test comparing group means.

Legends of Supplementary Movies (1 to 10)

Supplementary movie 1. Observation of the α-catenin-vinculin-VASP activity on single actin filaments in TIRF microscopy at 25 mM KCl in the presence or the absence of profilin. Time-lapse sequence showing actin polymerisation using 0.8 µM G-actin (10% Atto488-labelled) in fluorescence buffer (5 mM Tris pH 7.8, 200 µM ATP, 5 mM 1,4-diazabicyclo[2.2.2]octane (DABCO), 0.4% methylcellulose, 25 mM KCl, 1 mM $MgCl_2$, 200 µM EGTA, 20 mM DTT), either alone or supplemented with ΔMod, V1ab4, and VASP (1 µM:1 µM:0.6 µM). Conditions are shown in the absence (top) or presence (bottom) of 5 µM profilin. Scale bar: 20 µm.

Supplementary movie 2. Actin polymerisation in micropatterned surfaces coated with a mix of α-catenin-VASP or α-catenin-vinculin-VASP. Conditions: 1 µM actin (2% AlexaFluor647N-labelled) in fluorescence buffer (5 mM Tris pH 7.8, 200 µM ATP, 5 mM 1,4-diazabicyclo[2.2.2]octane (DABCO), 0.4% methylcellulose, 25 mM KCl, 1 mM $MgCl_2$, 200 µM EGTA, 20 mM DTT), supplemented with 5 µM profilin. Before adding actin, micropatterns were coated with either a mix of α-catenin ΔMod and Atto488-VASP (1:1, final concentration 1.6 µM; top panels) or with a mix of α-catenin ΔMod, V1ab4 and Atto488-VASP (1:1:1, final concentration 1.6 µM; bottom panels). Scale bar: 20 µm.

Supplementary movie 3. Observation of the α-catenin-vinculin-VASP activity on single actin filaments in TIRF microscopy at 100 mM KCl in the presence or the absence of profilin. Time-lapse sequence of 0.8 µM G-actin (10% Atto488-labelled) polymerisation in fluorescence buffer (5 mM Tris pH 7.8, 200 µM ATP, 5 mM 1,4-diazabicyclo[2.2.2]octane (DABCO), 0.4% methylcellulose, 100 mM KCl, 1 mM $MgCl_2$, 200 µM EGTA, 20 mM DTT), either alone or supplemented with ΔMod, V1ab4, and VASP (1 µM:1 µM:0.6 µM). Time-lapse images are acquired in the absence (top) or presence (bottom) of 5 µM profilin. Scale bar: 20 µm.

Supplementary movie 4. FRAP on actin networks formed on micropatterned surfaces coated with α-catenin–VASP or α-catenin–vinculin–VASP. Conditions: 1 µM actin (2% AlexaFluor647N-labelled) in fluorescence buffer (5 mM Tris pH 7.8, 200 µM ATP, 5 mM 1,4-diazabicyclo[2.2.2]octane (DABCO), 0.4% methylcellulose, 25 mM KCl, 1 mM $MgCl_2$, 200 µM EGTA, 20 mM DTT), supplemented with 5 µM profilin. Prior to actin addition, micropatterns were coated with either a mix of α-catenin ΔMod and Atto488-VASP (1:1, final concentration 1.6 µM, top panels), or with a mix of α-catenin ΔMod, V1ab4 and Atto488-VASP (1:1:1, final concentration 1.6 µM, bottom panels). FRAP parameters: rectangular regions (100 by 200 pixels) were selected and bleached using 30 iterative pulses of 20 milliseconds each with the 647 nm laser at 80% power. Pre-bleach acquisition was carried out for 10 minutes (one frame every 10 seconds), followed by a 25-minute recovery phase (one frame every 10 seconds), using the 647 nm laser. Scale bar: 20 µm.

Supplementary movie 5. Effect of capping protein (CP) on actin polymerisation induced by the α-catenin–vinculin–VASP machinery. Conditions: 5 µM actin (2% AlexaFluor647N-labelled) in fluorescence buffer (5 mM Tris pH 7.8, 200 µM ATP, 5 mM 1,4-diazabicyclo[2.2.2]octane (DABCO), 0.4% methylcellulose, 25 mM KCl, 1 mM $MgCl_2$, 200 µM EGTA, 20 mM DTT), supplemented with 20 µM profilin and increasing concentrations of capping protein (CP) at 0, 5, and 10 nM. Prior to actin addition, micropatterns were coated with ΔMod-V1ab4-Atto488-VASP (1:1:1, final concentration of 1.6 µM). Scale bar: 20 µm.

Supplementary movie 6. Actomyosin-dependent binding of vinculin (Vh-eGFP) to α-catenin. Conditions: 50 nM AlexaFluor568-labelled myosin II (5% labelled) in fluorescence buffer (5 mM Tris pH 7.8, 200 µM ATP, 5 mM 1,4-diazabicyclo[2.2.2]octane (DABCO), 0.4% methylcellulose, 25 mM KCl, 1 mM $MgCl_2$, 200 µM EGTA, 20 mM DTT), supplemented with an ATP regeneration mix (2 mM ATP, 2 mM $MgCl_2$, 10 mM creatine phosphate, 3.5 U/mL creatine kinase) and 100 nM Vh-eGFP, in the absence (top) or presence (bottom) of 2.4 µM actin (2% AlexaFluor647N-labelled) and 10 µM profilin. Prior to actin addition, micropatterns were coated with wild-type α-catenin at 10 µM. Scale bar: 20 µm.

Supplementary movie 7. Actomyosin-dependent binding of vinculin (V1ab4) to α-catenin. Conditions: 2.4 µM actin (2% AlexaFluor647N-labelled) in fluorescence buffer (5 mM Tris pH 7.8, 200 µM ATP, 5 mM 1,4-diazabicyclo[2.2.2]octane (DABCO), 0.4% methylcellulose, 25 mM KCl, 1 mM $MgCl_2$, 200 µM EGTA, 20 mM DTT), supplemented with an ATP regeneration mix (2 mM ATP, 2 mM $MgCl_2$, 10 mM creatine phosphate, 3.5 U/mL creatine kinase), 10 µM profilin, and 100 nM AlexaFluor568-labelled V1ab4, in the absence (top) or presence (bottom) of 50 nM myosin II. Prior to actin addition, micropatterns were coated with wild-type α-catenin at 10 µM. Scale bar: 20 µm.

Supplementary movie 8. Actomyosin-dependent assembly of the α-catenin-vinculin-VASP machinery. Conditions: 2.4 µM actin (2% AlexaFluor647N-labelled) in fluorescence buffer (5 mM Tris pH 7.8, 200 µM ATP, 5 mM 1,4-diazabicyclo[2.2.2]octane (DABCO), 0.4% methylcellulose, 25 mM KCl, 1 mM $MgCl_2$, 200 µM EGTA, 20 mM DTT), supplemented with an ATP regeneration mix (2 mM ATP, 2 mM $MgCl_2$, 10 mM creatine phosphate, 3.5 U/mL creatine kinase), 10 µM profilin, and 100 nM Atto488–VASP in all conditions. **Top row:** no V1ab4, actin alone (left) or actin with 50 nM myosin II (right). **Bottom row:** with V1ab4 (100 nM); actin alone (left) or actin with 50 nM myosin II (right). Prior to the addition of the actin mix, micropatterns were coated with α-catenin at 10 µM. Scale bar: 20 µm.

Supplementary movie 9. Effect of α-catenin ΔMod, V1ab4, and VASP on branched actin filaments. Single actin filaments observed in TIRF microscopy in the presence of 0.8 µM actin (10% Atto488-labelled), supplemented with the indicated combinations of 50 nM Arp2/3, 100 nM VCA, 1 µM ΔMod, 1 µM V1ab4 and 0.6 µM VASP. Scale bar: 20 µm.

Supplementary movie 10. Actin assembly resulting from the concomitant action of Arp2/3 and the actomyosin-dependent α-catenin–vinculin–VASP complex. Conditions: 2.4 µM actin (2% AlexaFluor647N-labelled) in fluorescence buffer (5 mM Tris pH 7.8, 200 µM ATP, 5 mM 1,4-diazabicyclo[2.2.2]octane (DABCO), 0.4% methylcellulose, 25 mM KCl, 1 mM $MgCl_2$, 200 µM EGTA, 20 mM DTT), supplemented with 10 µM profilin and 50 nM Arp2/3 complex. Before addition of the actin mix, micropatterns were coated either with N-WASP alone (100 nM; top panels) or with a combination of N-WASP (100 nM) and wild-type α-catenin (10 µM; bottom panels). **Top row:** In the absence (left) or presence (right) of 50 nM myosin II. For the top panels, no Atto488–VASP was added; the VASP channel is therefore black. **Bottom row:** Supplemented with 100 nM V1ab4 and 100 nM VASP–Atto488, in the absence (left) or presence (right) of 50 nM myosin II. Scale bar: 20 µm.

Supplementary data file

Excel spreadsheet including the data for all plots shown in the article’s figures, with each figure panel placed in a separate sheet
